# Supplementary material for: Accurately modeling RNase H-mediated antisense oligonucleotide efficacy
Source: Mol Ther Nucleic Acids. 2026 Jul 7;37(3):103004. doi: 10.1016/j.omtn.2026.103004 (PMC13427547; doi:10.1016/j.omtn.2026.103004)
Supplement: Document S1. Figures S1–S7 and Table S1 [file mmc1.pdf]

OMTN, Volume *37*

Supplemental information

Accurately modeling RNase H-mediated antisense oligonucleotide efficacy

Barney Hill, Maisie R. Jaques, Remya R. Nair, Nicola Whiffin, Matthew J.A. Wood, Stephan J. Sanders, Peter L. Oliver, Alyssa C. Hill, Carlo Rinaldi, and on behalf of the UPNAT Consortium

Supplemental Material

Supplemental Text

LLM Prompts for Data Extraction

To ensure the reproducibility of our data extraction pipeline, this section details the verbatim prompts used for table normalisation with gpt-5.

Prompt 1

```
## Task
Write a Python 3.11 script to convert OCR-extracted table XML data into a
structured CSV format.

## Output Format
Return a Script object with:
- `pyscript`: Complete Python conversion script as a string

## Function Requirements
- The Python script should contain a function `xml_to_csv(xml_str: str) ->
str`
- You do need to return if __name__ ... w/ example usage - just our
function.

## Technical Requirements
### Dependencies
- Uses only Python standard library + `re` module

### Column Name Rules
- Preserve meaning of original column headers in new csv. i.e "UTC
Untreated control group (%)" to "utc_untreated_control_group_pct" /
"Inhibition (%)" to "inhibition_pct"
- Make SQL-compatible: underscores for spaces, no dots, lowercase
- Hardcode column names (no need to dynamically generate)

### Data Handling
- Use "NA" for missing/empty cells
- Properly escape CSV values (quotes, commas, newlines)
- Use comma delimiter
- Sometimes   is used in the XML, this should be replaced with a
space in the CSV.

## Domain Context
- UTC = "Untreated Control" percentage - this is not inhibition
- Preserve scientific notation and decimal precision
- Don't interpret abbreviations unless obvious

## Quality control
- To ensure correct rows let's strip newspace and capitalise the sequence
column. It should have >=8 ATGC characters. If not skip the row.
```

```
## Preview of the input XML Structure (xml_str) - we will use the full
version as input for your script (do not return this in your output!):
Your goal is to produce a dataset of antisense-oligonucleotide sequences
and their inhibition percentages.
To do so you must stack the secondary_table with the primary_table using a
SQL command.

Required Columns in secondary_table:
1. ASO sequence (case insensitive)
2. One of:
   - inhibition/knockdown/reduction percentage
   -  UTC (Untreated Control) / RNA percentage

Transformation Rules:
- Numeric columns -> DOUBLE
- inhibition_percent =
    - Direct copy from inhibition/knockdown columns
    - 100 - UTC(%) for untreated control
- CONCAT two columns if ASO sequence is split (e.g.,sequence_part_one,
sequence_part_two)
- When using CAST be careful, some rows may not be castable to double,
hence use TRY_CAST.

Task:
1. Generate SQL:
   - Stack (INSERT INTO) secondary_table onto primary_table
   - Apply transformations as needed
   - **CRITICAL: Always use "secondary_table" as the table name in your
FROM clause**
   - **When referencing columns from secondary_table, use the exact column
names shown in the schema, including any special characters or numbers.**

Output Format:
- sql_command: string containing complete SQL command to stack the
secondary_table onto primary_table

Data:
primary_table:
Schema:
- aso_sequence_5_to_3 (VARCHAR): 5'-3' ASO nucleotide sequence
- inhibition_percent (DOUBLE): target inhibition percentage, range 0-100
```

Prompt 2

```
Your goal is to produce a dataset of antisense-oligonucleotide sequences
and their inhibition percentages.
To do so you must stack the secondary_table with the primary_table using a
SQL command.

Required Columns in secondary_table:
1. ASO sequence (case insensitive)
2. One of:
   - inhibition/knockdown/reduction percentage
   -  UTC (Untreated Control) / RNA percentage
```

```
Transformation Rules:
- Numeric columns -> DOUBLE
- inhibition_percent =
    - Direct copy from inhibition/knockdown columns
    - 100 - UTC(%) for untreated control
- CONCAT two columns if ASO sequence is split (e.g.,sequence_part_one,
sequence_part_two)
- When using CAST be careful, some rows may not be castable to double,
hence use TRY_CAST.

Task:
1. Generate SQL:
   - Stack (INSERT INTO) secondary_table onto primary_table
   - Apply transformations as needed
   - **CRITICAL: Always use "secondary_table" as the table name in your
FROM clause**
   - **When referencing columns from secondary_table, use the exact column
names shown in the schema, including any special characters or numbers.**

Output Format:
- sql_command: string containing complete SQL command to stack the
secondary_table onto primary_table

Data:
primary_table:
Schema:
- aso_sequence_5_to_3 (VARCHAR): 5'-3' ASO nucleotide sequence
- inhibition_percent (DOUBLE): target inhibition percentage, range 0-100
```

Supplemental Tables

Table S1. Description of the fields, data types, and definitions for each entry in the ASO Atlas dataset.

Field Name	Data Type	Description
aso_sequence_5_to_3	String	The nucleotide sequence of the ASO.
inhibition_percent	Float	The measured percentage of target RNA inhibition. Typically 0-100 although there are some values < 0 where upregulation was observed.
chemistry	Object	A object containing a list of all sugar and backbone modifications
custom_id	String	A string refering to the location of the referenced patent table file.
target_mrna	String	The name used to refer to the target mRNA.
target_gene	String	The HUGO gene name corresponding the the target_mrna
cell_line	String	The cell-line used for the screen.
dosage	Float	The dosage of the administered ASO in nM.
cells_per_well	Integer	The number of cells per well used in the screen.

Field Name	Data Type	Description
transfection_method	String	The method of introducing the ASO into the cells.

Supplemental Figures

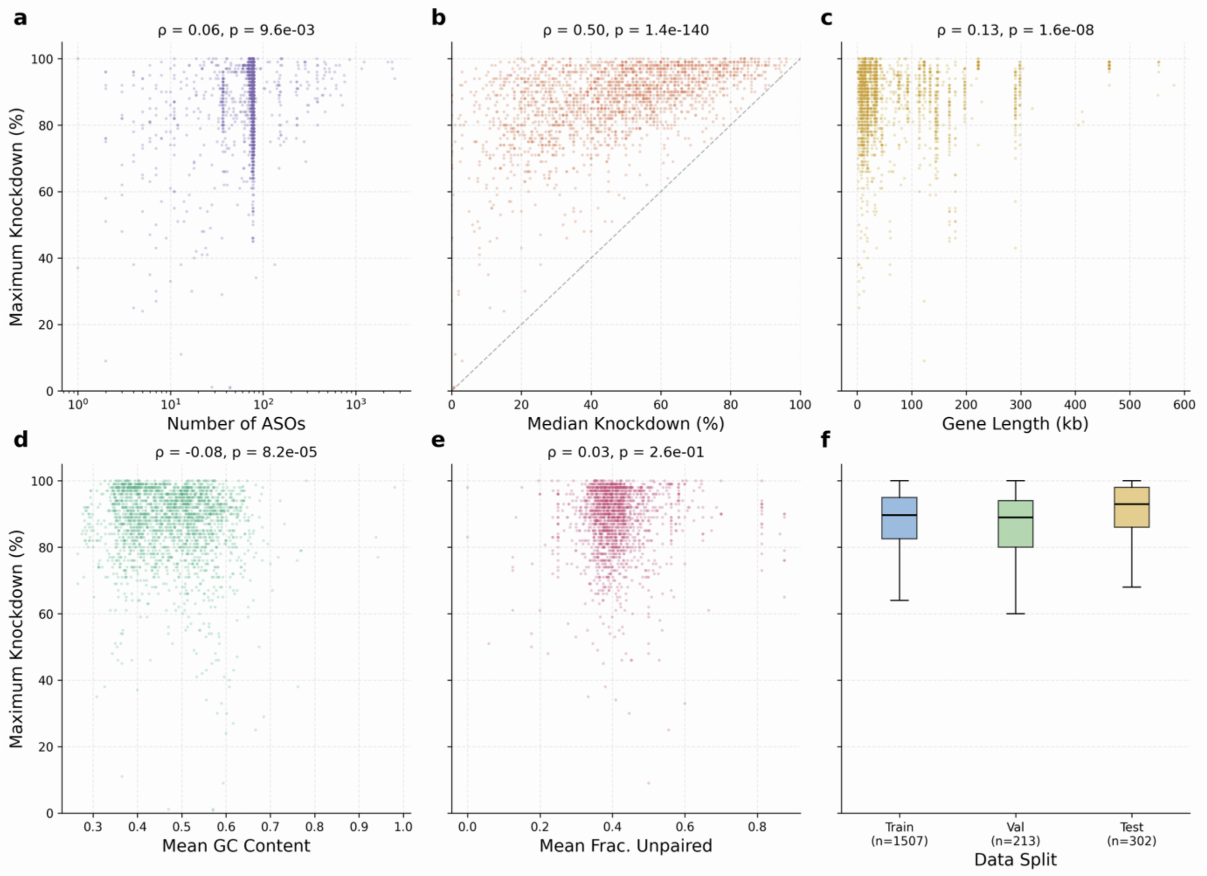

Figure S1. Influence of screen size, target properties, and data partitioning on maximum knockdown per screen. (a) Maximum knockdown as a function of the number of ASOs per screen (Spearman $\rho = 0.06, p = 9.6 \times 10^{-3}$). (b) Maximum versus median knockdown per screen ($\rho = 0.50, p = 1.4 \times 10^{-140}$). (c) Maximum knockdown versus gene length (genomic span; $\rho = 0.13, p = 1.6 \times 10^{-8}$). (d) Maximum knockdown versus mean ASO GC content ($\rho = -0.08, p = 8.2 \times 10^{-5}$). (e) Maximum knockdown versus mean fraction of unpaired nucleotides at the target site ($\rho = 0.03, p = 0.26$). (f) Distribution of maximum knockdown across training, validation, and test splits. Each point in (a–e) represents one screen. All correlations are Spearman rank coefficients.

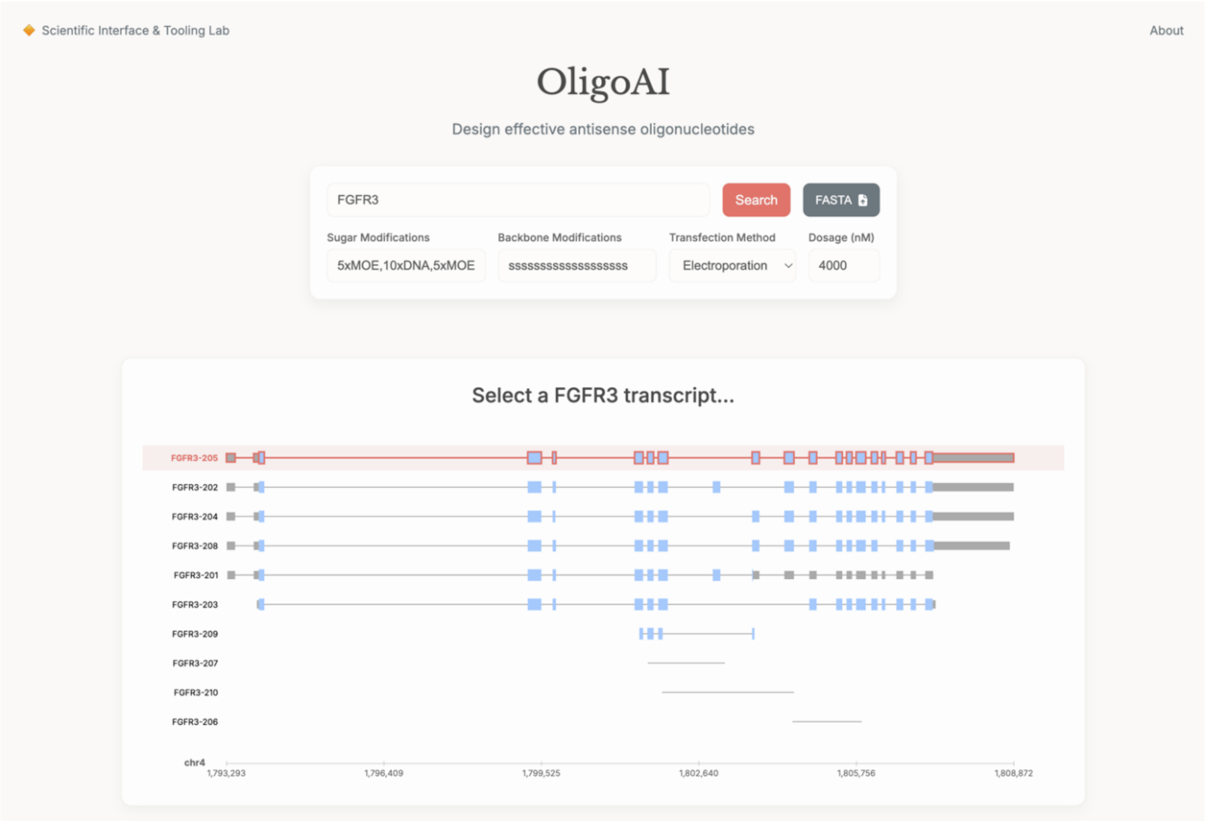

Figure S2. OligoAI online portal. Users can either supply a target RNA via FASTA file upload or select an Ensembl human transcript. When a target is specified, the OligoAI model processes the target RNA using serverless GPU inference. These results are returned to the user on the same page or via an email reminder and are available to download.

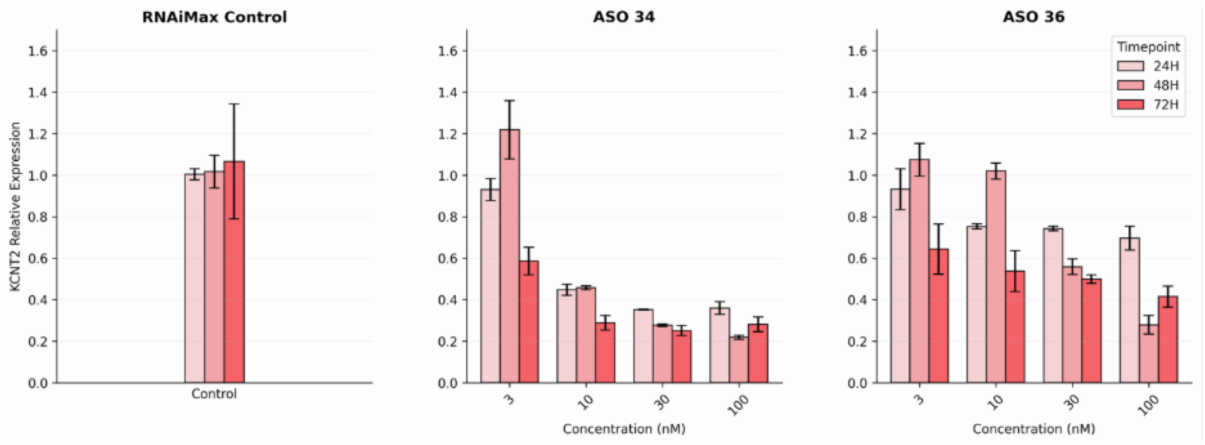

Figure S3. Dose- and time-dependent knockdown of *KCNT2* expression by selected ASOs in HeLa cells. Individual panels show the relative activity for three ASOs. Cells were treated at concentrations of 3, 10, 30 and 100 nM for 24, 48 or 72 hours. *KCNT2* mRNA levels were measured by quantitative RT-PCR and normalised to *HPRT*. Data represent mean $\pm$ SEM of three biological replicates per condition. Based on these results, 30 nM treatment for 48 hours was selected as the optimal condition. Data are shown relative to transfection reagent alone (RNAiMAX).

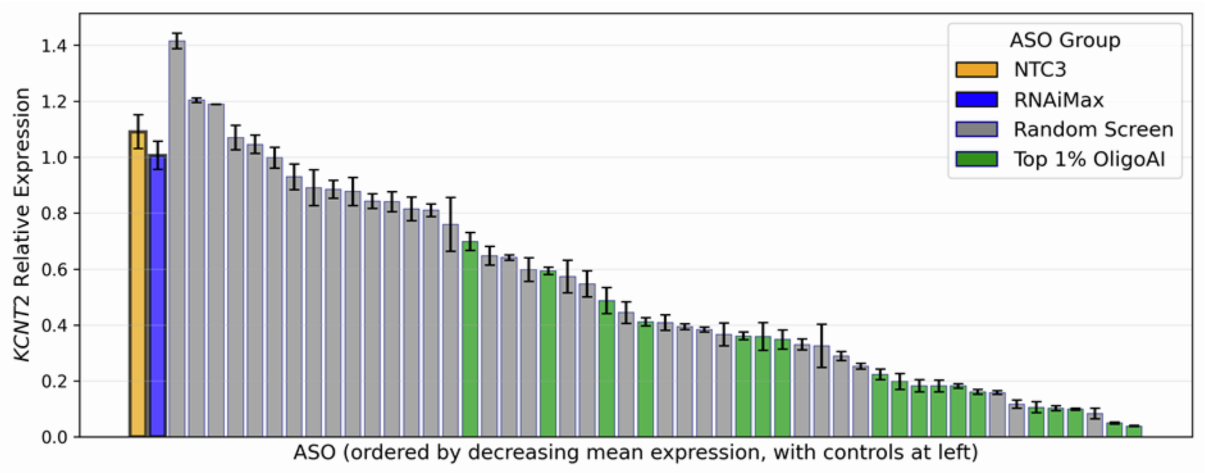

Figure S4. Bar plot showing relative *KCNT2* expression levels for all 50 screened ASOs ordered by decreasing mean expression (most effective knockdown on the right). HeLa cells were transfected with individual ASOs at 30 nM for 48 hours. Relative gene expression was determined by qRT-PCR with *HPRT* as the reference gene. Error bars represent SEM from three biological replicates. Bars are colored by ASO selection group: randomly selected ASOs (grey bars, standard screen, N = 32) and ASOs from the top 1% of OligoAI scores (green bars, predicted active, N = 18). The distribution demonstrates that OligoAI predictions correlate with experimental efficacy, with most top 1% ASOs showing greater knockdown activity (lower relative expression) compared to bottom 1% ASOs. Data are shown relative to transfection reagent alone (RNAiMAX) and a non-targeting control (NTC) ASO of matching chemical composition.

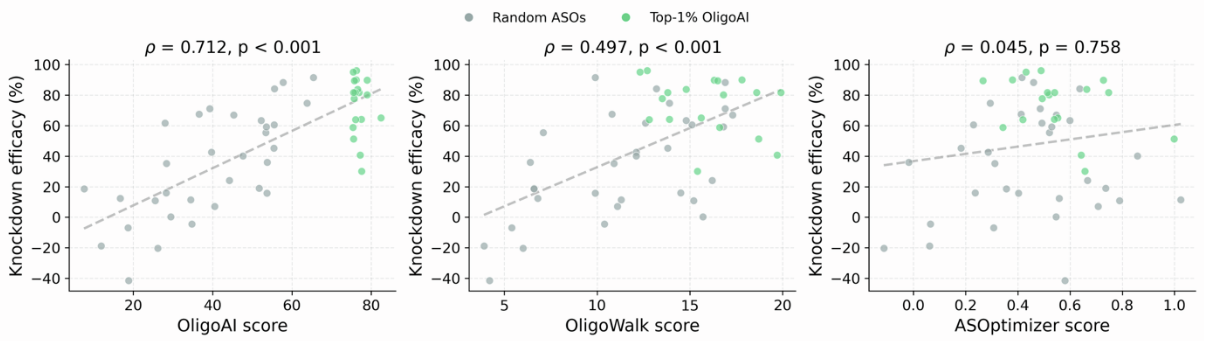

Figure S5. Correlation between predicted and measured *KCNT2* knockdown across methods. Scatter plots showing predicted score versus measured knockdown efficacy for all 50 experimentally tested *KCNT2* ASOs (32 randomly selected, 18 top-1% OligoAI-ranked), scored by OligoAI, OligoWalk, and ASOptimizer. Spearman rank correlation and associated P-values are shown above each panel. Trend lines are fitted by ordinary least squares.

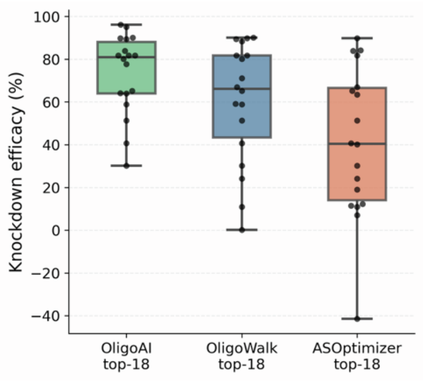

Figure S6. Comparison of top-18 ASO selections by each prediction method. Box-and-swarm plots showing the measured knockdown efficacy of the 18 ASOs each method would have prioritised from the 50 experimentally tested *KCNT2* ASOs. OligoAI's top-18 selections achieved the highest median knockdown (81%), compared with OligoWalk (66%) and ASOptimizer (40%).

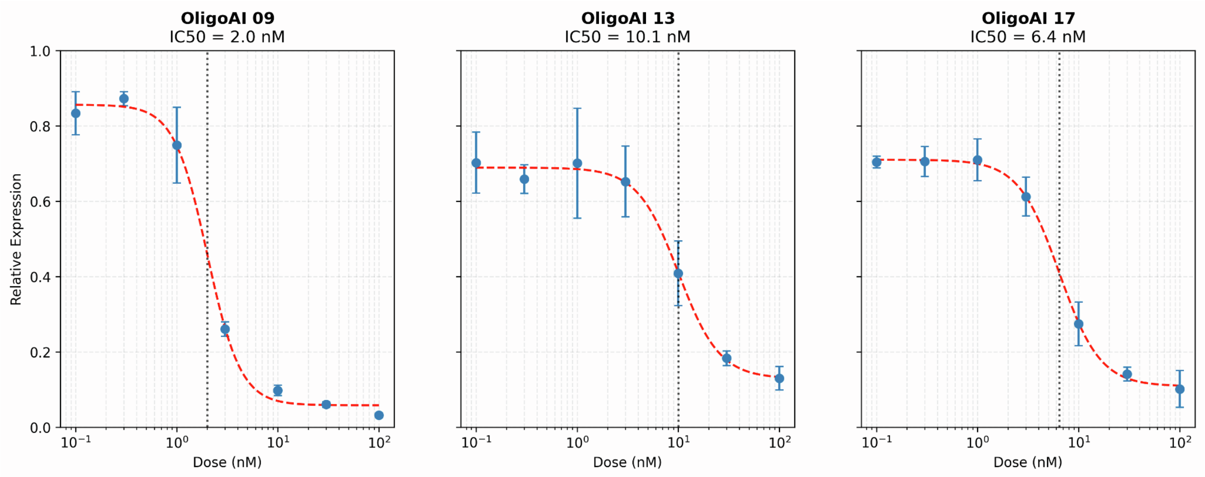

Figure S7. Individual dose-response curves showing relative *KCNT2* expression levels following ASO treatment in HeLa cells. Cells were transfected with ASOs at concentrations ranging from 0.1 nM to 1 μM and incubated for 48 hours. Each data point represents the mean $\pm$ SEM of three biological replicates. Relative gene expression was determined by qRT-PCR with *HPRT* as the reference gene. Red dashed lines show 4-parameter logistic curve fits (Hill equation). Black dotted vertical lines indicate calculated IC50 values. IC50 values demonstrate knockdown activity in the low nanomolar range.
