# Supplementary material for: Accurately modeling RNase H-mediated antisense oligonucleotide efficacy
Source: Mol Ther Nucleic Acids. 2026 Jul 7;37(3):103004. doi: 10.1016/j.omtn.2026.103004 (PMC13427547; doi:10.1016/j.omtn.2026.103004)
Supplement: Document S2. Article plus supplemental information [file mmc2.pdf]

Molecular Therapy
Nucleic Acids
Original Article

Accurately modeling RNase H-mediated antisense oligonucleotide efficacy

Barney Hill,[1,3,5] Maisie R. Jaques,[2] Remya R. Nair,[2] Nicola Whiffin,[3,4] Matthew J.A. Wood,[1,5,6] Stephan J. Sanders,[1,5,7,8] Peter L. Oliver,[2] Alyssa C. Hill,[1,5] Carlo Rinaldi,[1,5,6] and on behalf of the UPNAT Consortium

[1]Department of Paediatrics, University of Oxford, Oxford OX3 7TY, UK; [2]MRC Nucleic Acid Therapy Accelerator, Harwell Research and Innovation Campus, Didcot OX11 0FA, UK; [3]Big Data Institute and Centre for Human Genetics, University of Oxford, Oxford OX3 7LF, UK; [4]Broad Center for Mendelian Genomics, Program in Medical and Population Genetics, Broad Institute of MIT and Harvard, Cambridge, MA 02142, USA; [5]Institute of Developmental and Regenerative Medicine, IMS-Tetsuya Nakamura Building, Old Road Campus, Oxford OX3 7TY, UK; [6]Oxford Harrington Rare Disease Centre, IDRM, Old Road Campus, Oxford OX3 7TY, UK; [7]New York Genome Center, New York, NY 10013, USA; [8]Department of Psychiatry and Behavioral Sciences, UCSF Weill Institute for Neurosciences, University of California, San Francisco, San Francisco, CA 94178, USA

Antisense oligonucleotides (ASOs) are a powerful class of drugs with the potential to treat a wide range of human diseases. However, the prediction of ASO efficacy remains challenging, as large-scale and costly experimental screens are typically required to identify optimal candidates for a specific therapeutic target. To address this challenge, we compiled ASO Atlas, a database comprising 188,521 RNase H-mediated ASO sequences targeting 334 unique genes with corresponding knockdown efficacy measurements extracted from published patents. Using ASO Atlas, we trained OligoAI, a deep learning model capable of jointly modeling RNA target context, ASO sequence, sugar and backbone chemistries, and dosage to predict *in vitro* efficacy. We experimentally validated OligoAI by targeting *KCNT2*, achieving a 5.72-fold reduction in screening effort compared to random selection. ASO Atlas provides the first systematic resource to rigorously evaluate hypotheses regarding key parameters in ASO design, including sequence composition, chemical modifications, and target region selection. Both ASO Atlas and OligoAI have been made freely accessible through an online web-tool with the aim of facilitating the accelerated optimization of ASO design.

INTRODUCTION

Antisense oligonucleotides (ASOs) are short, synthetic, single-stranded nucleic acids designed to selectively hybridize with target RNA sequences and modulate gene expression.[1] ASOs can reduce target RNA levels or alter the splicing patterns of target RNA transcripts. The former is achieved by recruitment of RNase H, an enzyme that cleaves the RNA strand of the DNA-RNA heteroduplex formed between the ASO and its RNA target.[2,3] RNase H-dependent ASOs typically employ a "gapmer" design.[4] This design features a central gap of 8–10 DNA nucleotides, which are essential for RNase H recognition and cleavage, flanked by wings containing 3–5 nucleotides modified at the 2′ position of ribose (e.g., 2′-*O*-methoxyethyl, 2′-MOE; constrained ethyl, cEt), which increase binding affinity and stability[5] and enhance the ASO's drug-like properties.[3] The backbone is typically uniformly modified with the phosphorothioate (PS) linkage, which replaces one non-bridging oxygen atom of the natural phosphodiester (PO) linkage with a sulfur atom to confer nuclease resistance on the ASO. This strategy effectively balances the requirement for RNase H activity with the need for enhanced affinity and stability. Gapmer structures are often denoted by the lengths of the 5′ wing, DNA gap, and 3′ wing (e.g., 5-10-5).

Initially proposed as therapeutics over four decades ago,[6] ASOs utilizing gapmer designs have recently gained significant clinical momentum, with landmark approvals such as volanesorsen for familial chylomicronemia syndrome and inotersen for transthyretin amyloidosis, demonstrating their viability for treating rare genetic disorders.[7,8] As their safety profile becomes increasingly established, opportunities for treating ultra-rare genetic diseases continue to expand.[9–11]

Despite these clinical successes, the determinants of ASO efficacy remain poorly understood. Effective ASO design requires optimizing two key components: the pharmacophore, which is defined by the nucleotide sequence and dictates target specificity, and the dianophore, which includes the pattern of chemical modifications, which influence the drug-like properties.[5] However, predicting the combined impact of sequence and chemistry on efficacy remains a significant challenge. With gene transcripts containing thousands of potential ASO target sites, and there being many potential combinations of oligonucleotide length and chemical composition, current

Received 21 January 2026; accepted 6 July 2026;
https://doi.org/10.1016/j.omtn.2026.103004.

Correspondence: Barney Hill, Institute of Developmental and Regenerative Medicine, IMS-Tetsuya Nakamura Building, Old Road Campus, Oxford OX3 7TY, UK.
E-mail: barney.hill@merton.ox.ac.uk
Correspondence: Carlo Rinaldi, Institute of Developmental and Regenerative Medicine, IMS-Tetsuya Nakamura Building, Old Road Campus, Oxford OX3 7TY, UK.
E-mail: carlo.rinaldi@idrm.ox.ac.uk

Published by Elsevier Inc. on behalf of The American Society of Gene and Cell Therapy.
This is an open access article under the CC BY license (http://creativecommons.org/licenses/by/4.0/).

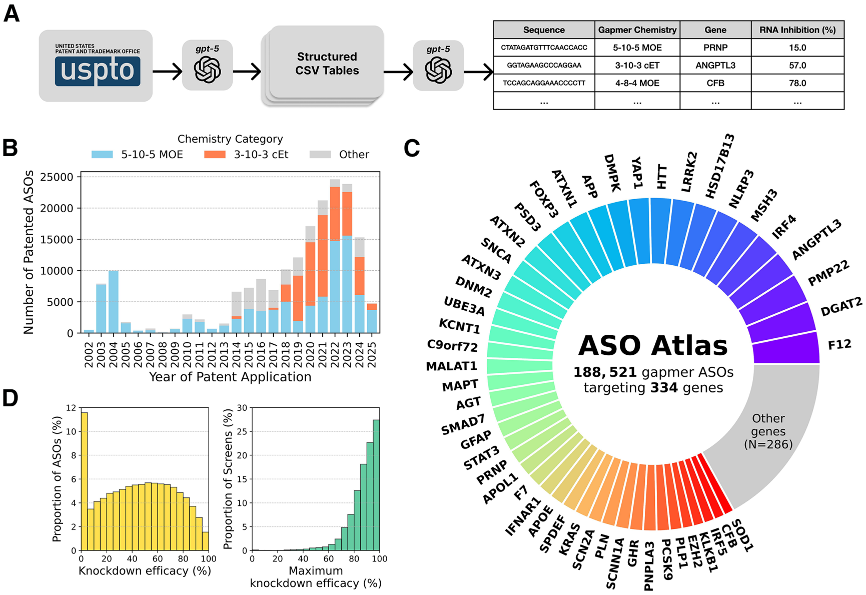

Figure 1. Construction and composition of the ASO Atlas dataset

(A) Pipeline for creating the ASO Atlas dataset, showing extraction of ASO data from USPTO patents using gpt-5 for initial structured table extraction, followed by annotation of sequence, chemistry, target gene, and knockdown measurements. (B) Temporal distribution of patented ASO sequences from 2001 to 2025, showing a peak in patented sequences in 2023. Blue represents 5-10-5 MOE modified ASOs, orange represents 3-10-3 cEt ASOs, and gray represents other chemistries. (C) Donut chart of ASO distribution across genes, demonstrating concentrated development efforts on select therapeutic targets. Major genes with ≥1,500 ASOs are labeled individually, while the "other genes" category encompasses 286 genes with <1,500 ASOs. (D) RNA knockdown distributions for all ASOs (left, yellow) showing a broad efficacy range including ineffective ASOs, and maximum knockdown per screen (right, green), indicating that a large proportion of screens achieve high knockdown with at least one ASO.

pre-clinical development strategies are heavily burdened by costly experimental screening efforts.

Current computational predictive models fail to capture the complex structure-activity relationships of ASOs,[12] and these approaches are further hampered by the lack of standardized, large-scale training datasets. Furthermore, existing computational tools are often constrained by proprietary data that cannot be independently evaluated. In this study, we present ASO Atlas, an extensive resource compiled from ASO screening data reported throughout the patent literature. Leveraging this comprehensive dataset, we have developed OligoAI, a machine-learning approach to jointly model how ASO sequence and chemical modifications influence *in vitro* efficacy, demonstrating a data-driven approach that enhances the identification of potent ASO candidates.

RESULTS

ASO atlas: A large-scale dataset of gapmer ASOs with corresponding *in vitro* efficacy measurements

The ASO Atlas comprises a diverse collection of 188,521 RNase H-mediated gapmer ASO sequences extracted from 417 United States Patent and Trademark Office (USPTO) patents published between 2001 and 2025. To construct this dataset, we implemented a multi-stage computational pipeline that began by manually labeling tables containing ASO knockdown efficacy data from patent applications made by IONIS Pharmaceuticals. We then employed a two-step large language model approach: OpenAI's gpt-5 converted raw XML patent tables into standardized CSV format using Python scripts, followed by systematic annotation of ASO sequences, chemical modifications, target genes, and quantitative efficacy measurements based on our dataset schema requirements with SQL (Figure 1A). This dataset contains nucleotide sequence composition, chemical modifications, target gene, and corresponding quantitative reverse-transcription polymerase chain reaction (RT-qPCR) RNA abundance measurements across multiple cell lines. Manual review of 100 randomly selected entries across 100 patents revealed no numerical transcription errors, and only minimal errors in chemical modification transcriptions (3% contained at least one error). The dataset features experiments conducted in a diverse range of cell types including A-431 (N = 44,855), HepG2 (N = 23,428), and SH-SY5Y (N = 18,192) and features 334 unique gene targets. The gene targets with the most ASOs were *F12* (N = 5,874), *DGAT2* (N = 5,326), and *PMP22* (N = 5,273) (Figure 1C). Among ASOs with chemistry annotations, 52.6% were 5-10-5 2′-MOE gapmers and 30.4% were 3-10-3 cEt gapmers (with varying PS backbone patterns), while the remaining proportion comprised diverse designs including alternative gapmer lengths (e.g., 4-10-4, 5-9-5) and mixed MOE/cEt chemistries. While the atlas spans from 2001 to 2025, we observed a notable increase in the volume of patented sequences in recent years (see 2018–2024, Figure 1B). The dataset exhibits a broad distribution of efficacy values (Figure 1D) with a median knockdown efficacy of 45.0%. Importantly, 17.4% of ASOs show <10% knockdown, indicating substantial inclusion of ineffective sequences. This representation across the full efficacy spectrum suggests that patent filings document comprehensive screening results, rather than selectively reporting successful candidates, thus providing a robust training signal for machine learning models (Figure S1).

Target site characteristics influence gapmer ASO efficacy

We systematically evaluated multiple factors influencing ASO efficacy, including nucleotide sequence composition, genomic location, regulatory element overlap, and RNA secondary structure. To identify position-specific nucleotide patterns associated with efficacy, we performed multivariate linear regression analysis for each nucleotide at each position along the ASO sequence, separately for 5-10-5 MOE

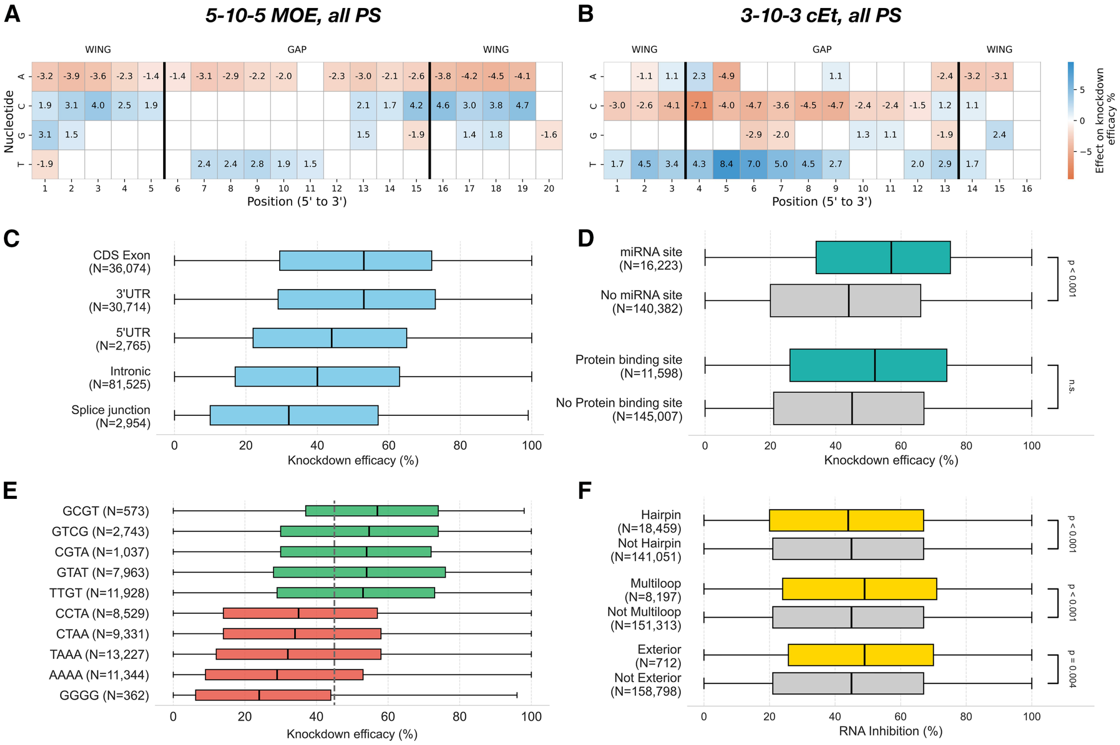

Figure 2. Analysis of ASO knockdown efficacy across genomic regions and sequence contexts

(A and B) Heatmaps of position-specific nucleotide effects on ASO efficacy for 5-10-5 MOE, all PS and 3-10-3 cEt, all PS chemistries, respectively. Effects from linear regression are shown only where significant after Bonferroni correction. Colors indicate effect magnitude on knockdown efficacy percentage, with blue representing positive effects and orange representing negative effects on efficacy. In (C)–(F), boxes show the interquartile range (IQR) with the median marked, and whiskers extend to the data range. (C) Comparison of efficacy distributions between ASOs overlapping different genomic regions. Regions are presented in order of median efficacy, and all group differences are significant (Mann-Whitney *U*, see materials and methods). (D) Comparison of efficacy distributions between ASOs overlapping miRNA binding sites as reported by TarBase and protein binding sites as reported by RBP-Tar.[13,14] (E) Box plots displaying top/bottom-5 median efficacy differences of 4-mer motifs (all significant after accounting for screen confounding with linear model, see materials and methods), with green bars showing positive effect motifs and red bars showing negative effect motifs. Dashed line represents overall median. (F) Comparison of efficacy distributions between ASOs overlapping RNA secondary structure elements, including hairpin, multiloop, and exterior loops, as annotated by ViennaRNA 2.0 using ±150 bp context around the ASO target.[15] The Mann-Whitney *U* test was used to compared the groups.

(all PS) and 3-10-3 cEt (all PS) chemistries. After Bonferroni correction, we identified 83 significant position-specific nucleotide effects that varied across chemistry types (Figures 2A and 2B). For example, cytosines showed opposite effects between chemistries: 13/20 positions were significantly positively associated with knockdown efficacy in 5-10-5 MOE ASOs, while 12/16 positions showed negative association in 3-10-3 cEt ASOs. The only pattern shared across both chemistry types was thymine showing positive association at positions 7–12 (5-10-5 MOE) and 1–9 (3-10-3 cEt) suggesting chemistry-dependent modulation of sequence composition effects.

Analysis of ASO efficacy across different genomic target region classifications revealed statistically significant variation in target knockdown ($p < 0.001$, Kruskal-Wallis test). We observed that ASOs targeting splice junction regions exhibited the lowest median knockdown efficacy (32.0% [IQR: 10.0%–57.0%]), followed by intron-targeting ASOs (40.0% [IQR: 17.0%–63.0%]) and 5′ untranslated region (UTR)-targeting ASOs (44.0% [IQR: 22.0%–65.0%]). In contrast, both 3′ UTR and exon-targeting ASOs demonstrated the highest median knockdowns (53.0% [IQR: 28.0%–73.0%] and 53.0% [IQR: 29.0%–72.0%], respectively). We note that higher efficacy is observed for loci present in both mature RNA transcripts and pre-mRNA (coding exons, 3′ UTR, and 5′ UTR) compared to those only present in pre-mRNA (splice sites and introns), (Figure 2C).

To further investigate the mechanisms underlying these regional differences, we first examined overlaps with microRNA (miRNA) and RNA-binding protein sites, given their enrichment in UTRs where we observed the varying efficacy (Figure 2D).[16] After accounting for regional variation in ASO efficacy with a multivariate linear regression analysis controlling for genomic region type, only miRNA binding site overlap ($\beta = 4.43\%$, $p < 0.001$) was identified as a significant predictor of efficacy.

Next, to identify sequence motifs that influence ASO knockdown efficacy, we analyzed all possible 4-mer nucleotide patterns while statistically controlling for variability between experimental screens. The "GGGG" motif showed the most pronounced

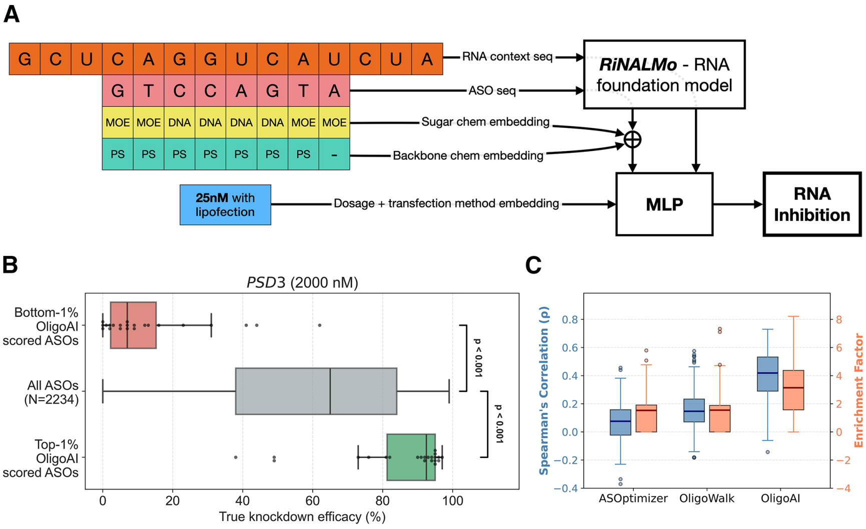

Figure 3. OligoAI model architecture and performance evaluation

(A) Architecture of OligoAI showing the integration of multiple feature modalities. ASO sequences and their target RNA context (±50 nucleotides) are encoded using the pre-trained RiNALMo-giga transformer model. Position-specific sugar modifications and backbone chemistry are encoded through learned embeddings and concatenated with RiNALMo sequence representations. The final prediction is generated by an MLP that integrates the pooled ASO representation, pooled context representation, and dosage-scaled transfection method information to predict percent knockdown. (B) Validation on a large held-out screen (*PSD3*: N = 2,234) showing that OligoAI-predicted top 1% ASOs achieve significantly higher knockdown than screen average, while predicted bottom 1% ASOs show significantly lower knockdown (one-sample Wilcoxon test). Box plots represent the IQR distribution. (C) Distribution of Spearman's correlation (ρ) and enrichment factor across N = 299 held-out test screens, comparing OligoAI performance to baseline methods (ASOptimizer, OligoWalk). Enrichment factor is the ratio of hit rate in the predicted top 10% to the baseline hit rate of 10%, where hits are defined as ASOs in the true top 10% by measured inhibition. Box plots represent the IQR distribution.

significant association; ASOs containing this sequence exhibited a markedly lower median knockdown efficacy (24.0%), a finding consistent with previous studies[17] (Figure 2E). Several other motifs, including "AAAA" (29.0%), "TAAA" (32.0%), "CTAA" (34.0%), and "CCTA" (35.0%), were also significantly associated with lower efficacy (all p < 0.001). In contrast, motifs such as "TTGT" (53.0%), "GTAT" (54.0%), "CGTA" (54.0%), "GTCG" (54.7%), and "GCGT" (57.0%) were significantly associated with higher efficacy.

Finally, we investigated the role of RNA secondary structure on efficacy based on overlap with ViennaRNA-predicted pre-mRNA secondary structure elements (using ±150 bp context).[15] After adjusting for screen-to-screen variability using a linear mixed model, ASOs targeting unpaired regions showed 8.5% higher knockdown efficacy compared to those targeting base-paired regions, indicating that target accessibility significantly influences ASO performance (p < 0.001). Predicted structure types showed distinct effects on efficacy. Hairpin structures were associated with decreased median knockdown efficacy (43.0% vs. 45.0%, N = 18,459 vs. 141,051, p < 0.001, Mann-Whitney U test), but multiloop structures enhanced performance (49.0% vs. 45.0%, N = 8,197 vs. 153,313, p < 0.001, Mann-Whitney U test). Exterior loop structures also improved efficacy (49.0% vs. 45.0%, N = 712 vs. 158,798, p = 0.0036, Mann-Whitney U test) (Figure 2F).

OligoAI: A deep learning model for ASO efficacy prediction

To leverage the sequence, structural, and positional determinants identified in ASO Atlas, we developed OligoAI (https://sitlabs.org/OligoAI), a transformer-based deep learning model that jointly encodes ASO nucleotide sequence, target RNA context, chemical modifications, dosage and transfection method to predict *in vitro* target RNA knockdown (Figures 3A and S2). The model takes the ASO sequence and its target pre-mRNA context (±50 nucleotides flanking the hybridization site), position-specific chemical modifications, including 2′-MOE and cEt sugar modifications, as well as PS and PO backbone linkages, and experimental parameters such as dosage and transfection method.

The architecture integrates these feature modalities through a multi-stage encoding process. ASO sequences and their target RNA contexts are independently encoded using RiNALMo-giga, a 650-million parameter bidirectional transformer pre-trained on 36 million non-coding RNA sequences, to capture local secondary structure accessibility and sequence context effects.[18] Chemical modifications are represented through learned embeddings and concatenated with the RiNALMo-encoded ASO sequence representations. These combined features are processed through a bottleneck network to learn cross-modal sequence-chemistry interactions. The resulting ASO and context representations are then globally pooled and integrated with dosage-scaled transfection method embeddings through a multi-layer perceptron to generate final efficacy predictions.

When evaluated across 299 held-out test screens, OligoAI achieved a median Spearman's correlation of 0.419 [IQR: 0.290–0.533] between predicted and measured knockdown efficacy values, with a 3.14 × enrichment factor for identifying high-performing ASOs (meaning the top 10% of OligoAI predictions contained 3.14 times more ASOs that were truly in the top 10% by measured knockdown efficacy compared to random selection) (Figure 3C). This substantially outperforms previous state-of-the-art thermodynamics-based models ASOptimizer (ρ = 0.076, 1.53 × enrichment) and OligoWalk (ρ = 0.147, 1.55 × enrichment), which rely primarily on hybridization energetics and target site accessibility.[12,19] Notably,

of the 185,585 PS-containing ASOs in the ASO Atlas, 31.0% (57,524) feature mixed PS/PO backbones, typically with PS being removed at selected wing positions. Mixed PS/PO ASOs similarly constituted 32.7% of the training set, indicating that reduced-PS designs are well represented. To assess generalization, we stratified the test screens by their dominant backbone pattern. On the 50 mixed PS/PO screens, OligoAI achieved a median Spearman's correlation of 0.520 [IQR: 0.446–0.606], compared with 0.393 [IQR: 0.274–0.495] on the uniform PS screens (Mann-Whitney *U*, $p = 7.0 \times 10^{-9}$), with identical enrichment factors of 3.14, showing that OligoAI generalizes robustly to reduced-PS designs.

To assess OligoAI's practical utility for identifying hits for *in vitro* gene screens with many possible sequence designs, we evaluated its performance on a large held-out screen targeting *PSD3* (N = 2,234 ASOs). These ASOs, derived from a single IONIS Pharmaceuticals patent (US20230167446A1), comprise 16-mer cEt 3-10-3 gapmers tested under uniform conditions (2,000 nM, gymnotic delivery), spanning the full 553 kb *PSD3* locus across intronic (76%), 3′ UTR (13%), exonic (10%), and splice junction (<1%) regions, with dense local sampling (22.6% of consecutive pairs separated by a single nucleotide) and a broad efficacy distribution (median 65%, IQR 37%–95%), consistent with comprehensive tiling rather than pre-filtering for efficacy. The model demonstrated robust discriminative ability: while the overall screen achieved a median knockdown efficacy of 65.0%, OligoAI's top 1% scored ASOs (N = 22) achieved a median knockdown efficacy of 92.5%, ($p < 0.001$, one-sample Wilcoxon test) (Figure 3B), highlighting OligoAI's potential to substantially reduce experimental screening burden in therapeutic ASO development.

Experimental validation of OligoAI prioritization in *KCNT2* screening

To test the utility of OligoAI in a real-world experimental screening setting, we generated a virtual library of 200,374 unique 20-mer gapmer ASOs targeting the Potassium Sodium-Activated Channel Subfamily T Member 2 (*KCNT2*), a gene associated with a rare developmental epileptic encephalopathy.[20,21] All possible 20-mers across the *KCNT2* locus were enumerated, excluding those overlapping repeat-masked regions, and sequences recurring at multiple genomic positions were de-duplicated at the first occurrence.

To confirm that OligoAI prioritizes the previously identified biologically relevant features, we examined whether the top 1% ranked *KCNT2* ASOs were enriched for genomic and sequence characteristics associated with efficacy. Top-ranked candidates showed significant enrichment for exonic targets (N = 118/2,006 in top 1% vs. N = 5,510/198,368 in remaining sequences; fold enrichment = 2.12, $p = 1.43 \times 10^{-13}$, Fisher's exact test), presence of top-5 sequence motifs (TTGT, GTAT, CGTA, GTCG, GCGT; N = 570/2,006 vs. N = 32,707/198,368; fold enrichment = 1.72, $p = 2.61 \times 10^{-40}$), and miRNA binding site overlap (N = 22/2,006 vs. N = 658/198,368; fold enrichment = 3.31, $p = 2.35 \times 10^{-6}$). Finally, proportion of unbound target RNA structure, defined as the unpaired fraction of nucleotides within the ASO binding sites in the predicted structure, showed little difference between groups (mean = 0.423 vs. 0.421; fold change = 1.00, p = 0.048, Mann-Whitney *U* test, structure predicted using ViennaRNA 2.0 using ±150 bp context around the ASO target[15]).

Two groups of ASOs were defined for *in vitro* testing: 32 sampled from the entire library and 18 sampled from the 1% of ASOs with the highest OligoAI scores. In both cases, random sampling was adopted to avoid selecting ASOs clustering along the same sites. To determine a suitable experimental dose for the ASO testing, a pilot experiment using two randomly chosen "active" ASOs was carried out by transfection in HeLa cells. After testing a range of doses and incubation times, 30 nM dosing for 48 h was selected as it provided the most suitable dynamic range of *KCNT2* knockdown by the "active" ASOs with no likely floor effects (Figure S3). In the subsequent complete library screening experiment, the relative *KCNT2* knockdown level was determined for all 50 ASOs in parallel, providing a ranking of *in vitro* efficacy (Figure S4). ASOs ranked in the top 1% by OligoAI achieved superior target knockdown efficacy (median = 81% [IQR: 64%–88%]) compared to randomly selected ASOs (median = 36% [IQR: 11%–62%]) (Mann-Whitney *U* test, $p = 1.1 \times 10^{-4}$). Retrospective scoring of all 50 experimentally tested *KCNT2* ASOs showed that OligoAI predictions correlated most strongly with measured knockdown (ρ = 0.712), followed by OligoWalk (ρ = 0.497) and ASOptimizer (ρ = 0.045) (Figures S5 and S6). These results demonstrate OligoAI's ability to effectively identify high-performing ASO candidates (Figure 4). Follow-up dose-response studies confirmed these results were reproducible, with IC50s in the low nanomolar range (Figure S7).

To quantify the practical screening efficiency gains, we performed bootstrap analysis (N = 10,000 iterations) to estimate the screening effort required for random selection to achieve equivalent performance to OligoAI's top candidates. Our analysis revealed that to match the median knockdown efficacy of our 18 top-scored ASOs, a standard random screening approach would require testing 103 ASOs (95% confidence interval, CI: 53–179). This represents a 5.72-fold reduction in required screening effort (95% CI: 2.94–9.94-fold), demonstrating substantial cost savings and accelerated candidate identification for ASO development workflows.

DISCUSSION

Our systematic analysis of 188,521 gapmer ASOs reveals key principles governing RNase H-mediated ASO efficacy and establishes a predictive framework for therapeutic design. The observed variation in efficacy across genomic regions, combined with secondary structure analysis, points to RNA accessibility as a central determinant of ASO performance. The reduced efficacy observed for hairpin-targeting ASOs compared to those targeting multiloop and exterior loop regions suggests that stable secondary structures present thermodynamic barriers to ASO hybridization. This interpretation is further supported by the positive association between miRNA binding site overlap and ASO efficacy, since miRNA target sites must be structurally accessible to enable miRNA binding, suggesting that ASOs similarly benefit from targeting accessible regions.[22] Together, these

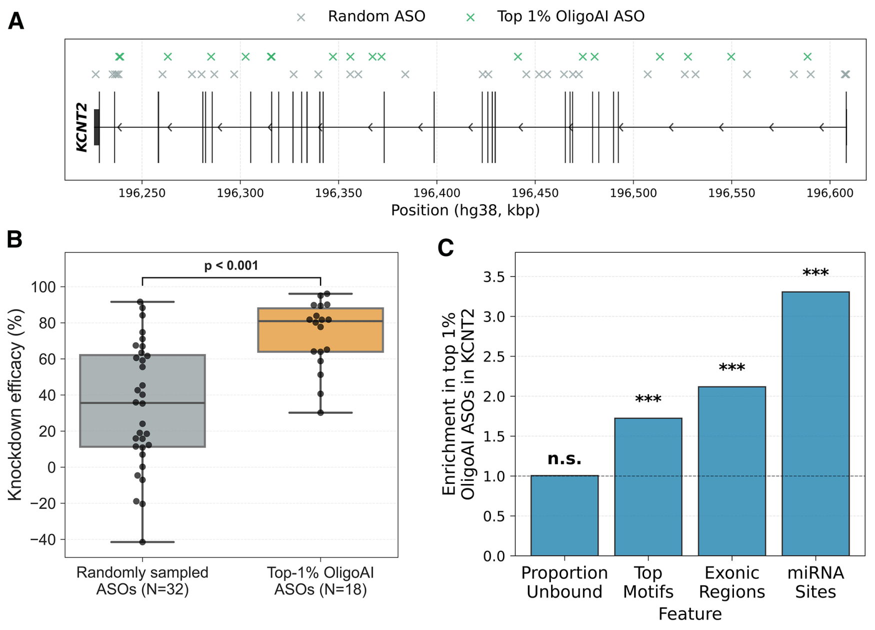

Figure 4. Experimental validation of OligoAI prioritization in *KCNT2* screening

(A) Schematic representation of the *KCNT2* transcript structure showing the genomic locations of ASOs sampled from two groups: randomly selected ASOs ($N = 32$), and top 1% OligoAI-ranked ASOs ($N = 18$). (B) Comparison of *KCNT2* knockdown efficacy across the ASO groups, expressed as relative target gene expression following transfection in HeLa cells. Top 1% OligoAI-ranked ASOs demonstrated significantly superior performance compared to randomly selected ASOs (Mann-Whitney U test, $p = 1.1 \times 10^{-4}$), with median relative expression levels of 0.19 versus 0.64, respectively. Box plots present the IQR distribution. (C) Bar plot showing the enrichment of biologically relevant features (exonic targeting, top-5 motifs, miRNA binding sites, and unbound RNA structure) within the top 1% ranked *KCNT2* ASOs compared to remaining sequences. Asterisks indicate statistical significance after Bonferroni correction (***$p < 0.001$; n.s., not significant).

findings indicate that successful ASO design must consider not only sequence complementarity but also the structural and genomic context of the target site.

ASO design has historically relied on heuristic trial-and-error approaches with limited datasets, with computational tools primarily focusing on thermodynamic parameters while largely ignoring the chemical modifications that define modern therapeutics.[23,24] Existing algorithms such as OligoWalk predict hybridization stability based on nearest-neighbor thermodynamics but fail to account for the PS backbones and sugar modifications (2′-MOE, cEt) that comprise the majority of clinical ASO candidates.[19] More recently, ASOptimizer has emerged as a two-component framework that addresses both sequence selection and chemical modification optimization separately.[12] Its sequence engineering module employs a linear regression model incorporating three thermodynamic features: target site binding free energy, off-target binding potential, and secondary structure accessibility. ASOptimizer also includes a separate chemistry optimization module based on graph neural networks that optimizes modification patterns given a fixed sequence. However, this bifurcated approach has notable limitations. Our systematic analysis revealed strong interactive effects between sequence composition and chemistry type: position-specific nucleotide associations with efficacy were almost entirely different between 5–10–5 MOE and 3-10-3 cEt chemistries (Figures 2A and 2B), with only thymine showing consistent patterns across both. These chemistry-specific sequence preferences demonstrate that nucleotide composition and chemical modifications do not act independently but rather likely interact to determine ASO efficacy. By treating sequence selection and chemistry optimization as independent problems, ASOptimizer cannot capture these complex, non-linear interactions between nucleotide sequence, chemical modifications, and target RNA structure that govern ASO efficacy. In our comparative evaluation across 299 held-out screens, ASOptimizer's sequence module achieved only modest predictive performance (Spearman $\rho = 0.076$), substantially lower than OligoAI ($\rho = 0.419$). OligoAI's improved performance likely stems from several key architectural differences including jointly modeling sequence and chemistry rather than treating them as independent optimization problems and leveraging a pre-trained RNA language model (RiNALMo) which has been shown to perform across various RNA modeling tasks.

While OligoAI represents a significant advance in ASO design prediction, several important limitations highlight areas for future development. First, the current model is limited to predicting RNase H-dependent ASOs. For many targets, this modality presents a large sequence design space, providing both greater opportunity for model training and a stronger need for computational optimization to guide experimental screening efforts. Additionally, our reliance on automatic extraction of patent-derived data represents a constraint, with LLM-based annotation introducing chemistry annotation errors in 4% of cases. This is a tolerable rate for machine learning approaches but with scope for future improvement. Finally, future extensions to the OligoAI platform will incorporate *in vivo* efficacy data, off-target effects, and toxicity assessments, all critical factors that ultimately determine therapeutic success.

In summary, we have created the ASO Atlas, the largest publicly available database of ASOs with experimentally validated efficacy data, comprising 188,521 gapmers targeting 334 genes. Through systematic analysis of this resource, we generated critical insights into the sequence, positional, and chemical modification features that govern ASO performance *in vitro*, revealing that exonic targets demonstrate

Table 1. Antisense oligonucleotide modifications present in ASO Atlas

Modification type	ASO count	Unique patents
PS Backbone	185,585	409
2′-MOE	126,620	391
cEt	77,516	53
Any	188,521	417

superior efficacy. We translated these insights into OligoAI, a deep learning prediction tool validated through holdout screens and experimental *KCNT2* testing, achieving a 5.72-fold reduction in experimental screening burden. By making both the ASO Atlas database and OligoAI prediction tool freely available to the research community, we provide resources that will substantially reduce the cost and time associated with ASO development, accelerating the translation of antisense therapeutics from target identification to clinical application. When combined with emerging experimental platforms such as patient-derived organoid screening systems,[25] this work enables more rapid progression toward personalized ASO therapeutics, ultimately enhancing the accessibility of precision medicine approaches for patients with rare genetic disorders.

MATERIALS AND METHODS

Dataset collection and processing

Patent selection and initial data extraction

We constructed ASO Atlas by mining the USPTO Bulk Data Storage System (BDSS) for ASO efficacy data from 2001 to 2025. After filtering for patents published by either ISIS or IONIS Pharmaceuticals, we manually annotated tables containing knockdown efficacy data. From these tables, we also included the preceding five paragraphs as contextual information.

Table structure normalization and validation

We developed a multi-stage natural language processing pipeline to convert unstructured XML patent tables into standardized formats. The full prompts provided to each model are detailed in supplemental information, and the final dataset conforms to a structured schema, which is fully defined in Table S1. For table structure recognition, we employed OpenAI's gpt-5 with verbosity = "low" and reasoning = "low" to create a Python script to convert the raw XML table into normalized CSV format.[26] Subsequently, we performed schema validation using gpt-5 to generate SQL commands that concatenate the ASO sequence information and quantitative efficacy measurements (either inhibition percentage or percentage of untreated control [UTC]) across tables.

Chemistry annotation and sequence characterization

For each validated entry, we implemented comprehensive annotation across three main stages. First, we used gpt-5 to systematically extract and standardize chemical modification patterns from patent text through chemistry annotation. Our annotation framework captured three types of chemical modifications (PS backbone, 2′-MOE sugar, and cEt sugar), as well as unmodified backbone (PO) and fully unmodified DNA sequences. For each ASO, we recorded both modification types and their precise positions along the sequence, with the model being provided with the five previous text paragraphs and row-specific data for this task. Manual review of 100 randomly selected entries revealed a 3% error rate in chemical modification transcriptions, with errors including the exclusion of mC modifications and a 4-9-4 gapmer being incorrectly recorded as a 5-7-5. These errors were manually corrected prior to downstream analysis. Second, we performed target information extraction using gpt-5 to annotate entries with metadata including target RNA name, HGNC (HUGO gene nomenclature committee) gene symbol, experimental cell line, ASO dosage (nM), cell density, and transfection method extracted from the five previous text paragraphs. Finally, we conducted genomic target mapping by mapping each ASO sequence to its target genomic location using the Ensembl 110 human genome assembly.[27] For each target gene, we selected the Ensembl canonical transcript, and ASO target sites were identified by searching for reverse complementary sequence matches between the ASO sequence and the pre-mRNA transcript.

Dataset quality assurance

We implemented rigorous quality control measures throughout the curation process. For sequence filtering, we restricted our dataset to ASOs with sequence lengths between 12 and 30 nucleotides and removed tables with duplicate sequence-inhibition pairs to prevent data redundancy. To ensure extraction accuracy, we performed manual verification by cross-referencing 100 randomly selected entries across 100 different patents. No errors were detected in ASO sequences and inhibition values in our validation sample, with a 97% accuracy rate in chemistry annotation. Regarding model selection rationale, we employed gpt-5 for annotation tasks based on benchmarking that demonstrated superior task accuracy and cost-effectiveness compared to alternative models such as gpt-5-mini. The final curated dataset comprises 188,521 ASOs from 417 patents, with comprehensive annotation of nucleotide sequences, chemical modifications, target genes, and experimental conditions (Table 1).

Position-wise nucleotide association testing

For each position along the ASO sequence and each nucleotide (A, C, G, T), we constructed a linear regression model with knockdown percentage as the dependent variable. The independent variables included: (1) a binary indicator for the presence of the specific nucleotide at the given position, (2) target gene identity, (3) transfection method, and (4) dosage (nM). This approach controlled for confounding effects of experimental conditions while isolating the contribution of each position-nucleotide combination to ASO efficacy.

Position-nucleotide combinations with insufficient variation (fewer than 5 ASOs containing or lacking the nucleotide at that position) were excluded from analysis. For each regression model, we extracted the coefficient and p value corresponding to the nucleotide indicator variable. To account for multiple hypothesis testing across all position-nucleotide combinations, we applied Bonferroni correction, with statistical significance defined as corrected $p < 0.05$.

Comparison of knockdown efficacy by genomic target region

ASO target regions were classified hierarchically into mutually exclusive categories with the following priority: 5′ UTR, 3′ UTR, splice junction (overlapping an exon-intron boundary), CDS (coding sequence) exonic and intronic as defined by Ensembl 110 canonical transcripts. ASOs overlapping miRNA or protein binding sites were defined by at least 10 nucleotides shared between the ASO target region and experimentally validated binding sites to reduce false positives. miRNA binding site regions were sourced from TarBase v.9 using PAR-CLIP (photoactivatable ribonucleoside-enhanced crosslinking and immunoprecipitation) or HITS-CLIP (high-throughput sequencing of RNA isolated by crosslinking immunoprecipitation) data,[13] while protein binding sites were obtained from RBP-Tar using eCLIP data ($p < 0.05$, Bonferroni-corrected for 515,644 sites).[14]

Comparisons of efficacy across different genomic target regions (5′ UTR, Splice Junction, Intron, Exon, 3′ UTR) utilized the Kruskal-Wallis H-test for an overall assessment of differences among the five region types. Following a statistically significant result, post hoc pairwise comparisons between specific region types were performed using Dunn's test, with p values adjusted for multiple comparisons using the Bonferroni correction method. All statistical analyses were performed using Python (v.3.10) with the SciPy library (v.1.15.1)[28] for the Kruskal-Wallis and Mann-Whitney U tests.

Motif analysis

To identify ASO motifs associated with knockdown efficacy while controlling for experimental batch effects, we performed a systematic analysis of all 256 possible 4-mer combinations. The motif length was chosen to balance statistical power and specificity, and it aligns with the length used in previous ASO sequence analyses.[17] For each motif, a multiple linear regression model was fitted to predict knockdown percentage based on the motif's presence, with the experimental screen (custom_id) included as a categorical covariate to account for screen-to-screen variability. To ensure adequate statistical power, only motifs present in at least 15 sequences were analyzed. We applied a strict Bonferroni correction for multiple comparisons, establishing a significance threshold of $p < 0.05/256$ ($\approx 1.95 \times 10^{-4}$). The direction of a motif's effect (positive or negative) was determined from its regression coefficient, and all motifs presented in this study were significant under these criteria.

RNA secondary structure analysis

For each ASO, we predicted the local RNA secondary structure of the target pre-mRNA using ViennaRNA 2.0.[15] A context window of 150 nucleotides around the ASO binding site was extracted from the transcribed pre-mRNA sequence (gene locus genomic sequence on the coding strand, including introns; GRCh38 primary assembly, Ensembl release 110) and folded using the minimum free energy (MFE) algorithm. Each nucleotide position in the predicted dot-bracket structure was classified as either paired (stem) or unpaired using ViennaRNA's element string annotation. Unpaired positions were further assigned to one of four loop types: hairpin loops (unpaired regions enclosed by a single base pair with no internal stems), interior loops (unpaired regions flanking a single internal stem, including bulges), multiloops (unpaired regions at junctions connecting two or more stems), and exterior loops (unpaired regions not enclosed by any base pair).

To quantify the effect of target site accessibility on ASO efficacy, we computed the fraction of unpaired nucleotide positions within each ASO binding region and fitted an ordinary least squares (OLSs) regression of knockdown efficacy on this continuous fraction, with experimental screen included as a categorical covariate to control for screen-to-screen variability. Each ASO target site was also assigned a dominant structure type, defined as the most frequent loop type among its unpaired positions. For each structure type, we compared the knockdown efficacy of ASOs with that dominant type against all remaining ASOs using Mann-Whitney U tests with Bonferroni correction for multiple comparisons.

Computational models for ASO efficacy prediction

Deep learning model architecture

We implemented OligoAI, a transformer-based architecture that leverages the pre-trained RNA language model RiNALMo to predict ASO efficacy. The model processes multiple input modalities to capture both sequence and experimental context.

- **Sequence representations**: ASO sequences and their target RNA context (ASO binding site ±50 flanking nucleotides) are encoded using RiNALMo-giga, a pre-trained bidirectional transformer model specialized for RNA sequences. Representations are extracted from the final transformer layer, yielding 1,280-dimensional embeddings per nucleotide position. The ±50 nucleotide context window was selected to capture local RNA secondary structure elements that determine ASO binding site accessibility, consistent with established approaches for local structure prediction,[29,30] while maintaining computational tractability for the transformer architecture.
- **Chemistry track**: Position-specific sugar modifications (MOE, cEt, DNA) are encoded through learned embeddings (16 dimensions).
- **Backbone track**: PS or PO linkages are represented through separate embeddings (8 dimensions).
- **Experimental conditions**: Transfection method (electroporation, gymnosis, lipofection, other) is encoded through learned 4-dimensional embeddings and element-wise multiplied by log-transformed dosage (log1p) to capture method-specific dose-response relationships.

The architecture integrates these features through a multi-stage process. First, ASO sequence representations from RiNALMo (1,280-dim) are concatenated with chemistry (16-dim) and backbone embeddings (8-dim).[18] These combined features (1,304-dim total) pass through a bottleneck network (linear 1,304-dim to 128, ReLU, dropout $p = 0.2$, linear 128-dim to 1,280, ReLU) to learn cross-modal representations.[31] Both ASO and context representations are then processed through global pooling layers that project to 64

dimensions while handling variable-length sequences via masked mean pooling. The final prediction is made by a 3-layer MLP with 128 hidden dimensions that combines the pooled ASO representation (64-dim), pooled context representation (64-dim), and method-scaled dosage embedding (4-dim) through fully connected layers with ReLU activations and dropout ($p = 0.3$).

Training and evaluation methodology

The model was trained to directly predict percent knockdown values using a mean squared error (MSE) regression objective. Target knockdown values were standardized (zero mean, unit variance) using a StandardScaler fitted on the training set, with predictions inverse-transformed to the original scale for evaluation. We initialized the model with pre-trained RiNALMo-giga weights and employed a gradual unfreezing schedule during fine-tuning to preserve learned RNA representations while adapting to the ASO efficacy prediction task. At epoch 0, only randomly initialized components were unfrozen (prediction head, chemistry embedder, backbone embedder, transfection method embedder). At epoch 3, we unfroze the pre-trained RiNALMo final layer normalization and transformer blocks 6–39. OligoAI was optimized using the Adam optimizer with a learning rate of 5×10^{-5}, weight decay of 0, and a linear learning rate schedule decaying to 5×10^{-6} (end factor = 0.1) over the total training steps.[32] Training proceeded for 10 epochs with a batch size of 64 samples, employing gradient clipping (max norm = 0.5) to ensure stable convergence. The model was trained with mixed precision (16-bit) on NVIDIA L40S GPUs (48 GB memory) to improve computational efficiency. The final model was selected based on the epoch achieving the lowest validation set MSE.

We evaluated the model using an 80/10/10 train/validation/test split, with stratification performed at the patent level to prevent data leakage between related ASO sequences. This patent-level splitting ensures that all ASOs from a given patent appear in only one split, addressing the tendency for patents to contain structurally and functionally related oligonucleotides. This yielded 134,948/18,309/25,367 ASOs across 1,507/213/302 screens from 295/36/38 non-overlapping patent groups in the train/validation/test sets, respectively. The validation set was used solely for model selection based on lowest MSE. Gene-level separation was not enforced; of the 31 genes in the test set, 25 are completely absent from training, while 6 (*ACAT2*, *C9orf72*, *GCGR*, *GHR*, *LRRK2*, *MALAT1*) appear in both splits from different patents under different experimental conditions. Sequence-level leakage was negligible, with only 62 of 22,245 unique test sequences (0.28%) appearing in the training data. Model performance was assessed using multiple metrics: coefficient of determination (R^2), mean absolute error (MAE), root mean squared error (RMSE), and Spearman's rank correlation (ρ) calculated per experimental screen and averaged across screens. Enrichment factor was defined as the ratio of the hit rate in the predicted top 10% to the baseline hit rate of 10%, where hits were defined as ASOs in the true top 10% by measured knockdown.

Model comparisons

To compare the relative performance of OligoAI, we used ASOptimizer and OligoWalk.[12,19] Specifically, from ASOptimizer we implemented the linear regression model from the sequence engineering module with the published regression parameters: $\hat{y} = a_0 + a_1x_1 + a_2x_2 + a_3x_3$, where $a_0 = -1.077$, $a_1 = -0.037$, $a_2 = 0.019$, $a_3 = 1.422$, x_1 represents the target Gibbs free energy change calculated by miRanda,[33] x_2 represents the mean of the top-10 off-target Gibbs free energy calculated against canonical protein coding pre-mRNA transcripts by miRanda,[31] and x_3 represents the secondary structure accessibility (ratio of unhybridized nucleotides) calculated by mFold.[29] From OligoWalk, we used the oligowalk_overall metric, which incorporates multiple thermodynamic terms including duplex stability (ΔG_{duplex}), target mRNA secondary structure stability ($\Delta G_{target\_structure}$), oligo self-structure ($\Delta G_{intra\_oligomer}$), and the oligo-oligo dimer ($\Delta G_{inter\_oligomer}$) to predict overall binding affinity.

Bootstrap estimation of screening efficiency

To quantify the screening effort required for random selection to match OligoAI's performance, we performed a bootstrap analysis with 10,000 iterations (random seed = 42). The resampling pool consisted of the 32 randomly selected ASOs only; the 18 OligoAI-ranked ASOs defined the performance target but were excluded from the pool. In each iteration, ASOs were drawn from the pool with replacement sequentially. After each draw, once 18 or more values had accumulated, we evaluated whether the median of the top 18 values (lowest relative expression) was less than or equal to the observed median relative expression of the 18 OligoAI-ranked ASOs (0.19). The iteration terminated at the first draw for which this criterion was met, and the total number of draws was recorded. The required screening effort was estimated as the median number of draws across all 10,000 iterations, with 95% CIs derived from the 2.5th and 97.5th percentiles. The fold reduction in screening effort was calculated by dividing the estimated number of random ASOs by 18.

To assess sensitivity to pool size, we repeated this procedure on the held-out *PSD3* screen ($N = 2,234$ ASOs), using OligoAI's predicted top 1% as the performance target. The *PSD3*-derived estimate (4.89-fold reduction) was consistent in magnitude with the *KCNT2* estimate (5.72-fold), suggesting that 32 random ASOs are sufficient to capture the approximate scale of the screening efficiency gain, with the concordance between the two independent estimates providing additional confidence despite the wide CI for the *KCNT2* analysis (95% CI: 53–179 ASOs, 2.94–9.94-fold).

Cell culture, ASO treatment, and gene expression analysis

HeLa cells were grown on 96-well plates (Thermo Scientific, 167008) at a seeding density of 10,000 cells per well in 100 μL media (DMEM with GlutaMAX (Gibco, 61965026), 10% fetal bovine serum (FBS; Gibco, A3840402) and 1% Penicillin Streptomycin (Gibco, 15070063). The following day, cells were treated with individual ASOs at the doses indicated. The media was replaced with 100 μL

fresh media before dosing and 20 μL of transfection mix (0.15 μL Lipofectamine RNAiMAX (Thermo Scientific, 13778150) with the ASO dose in OptiMEM (Gibco, 31985062) media). For the initial quantitative (q)RT-PCR assay optimization, ASOs were dosed for 24, 48, or 72 h at 3, 10, 30, or 100 nM. For the ASO library screen, ASOs were dosed for 48 h at 30 nM. Three biological replicate wells were used for each treatment. Controls included untransfected cells (OptiMEM), RNAiMAX transfection with no ASO, and a non-targeting control with a matching 5-10-5 2′-MOE gapmer design containing PS bonds throughout and 5′methyl-C/dC bases (5′-AGTCG CACACGTCTATACGC-3′). For dose-response analysis, a log10 dosing scale was used from 0.1 to 100 nM for 48 h as described previously. ASOs were synthesized by ATDBio or Integrated DNA Technologies (IDT) with QC by high-performance liquid chromatography (HPLC) and liquid chromatography-mass spectrometry (LC-MS).

After ASO dosing, total RNA was extracted using the MagMAX mirVana Total RNA Isolation Kit (Applied Biosystems, A27828). RNA concentration was measured using the Qubit RNA High Sensitivity Assay Kit (Invitrogen, Q32855) and cDNA synthesis was carried out using the High-Capacity cDNA Reverse Transcription Kit (Applied Biosystems, 4368813). Quantitative reverse-transcription PCR (RT-PCR) was performed using Fast SYBR Green Master Mix (Applied Biosystems, 4385618) on a CFX Opus 384 Real-Time PCR System (Bio-Rad). The following cycling conditions were used: enzyme activation at 95°C for 20 s, followed by 40 cycles of denaturation at 95°C for 3 s, annealing and extension at 60°C for 30 s. The RT-qPCR primer sequences were as follows: 5′-GTGCAGACACTCTTCAGGTTG-3′ and 5′-AGCCTCTCTCCCGTTCTTTC-3′ for *KCNT2*; 5′-AGT TCTGTGGCCATATGCTTAGTAG-3′ and 5′-AAACAACAATCC GCCCAAAGG-3′ for the reference gene *HPRT*. RT-qPCR data were analyzed using the comparative Ct ($\Delta\Delta Ct$) method: the Ct values of the target gene were normalized to the Ct values of the reference gene. Relative gene expression levels were calculated using the formula $2^{-\Delta\Delta Ct}$. All reactions were performed in technical triplicates per biological replicate and the average *Ct* values used for analysis.

DATA AND CODE AVAILABILITY

The ASO Atlas dataset scripts and OligoAI training code are available at https://github.com/barneyhill/aso_atlas and https://github.com/barneyhill/OligoAI. The trained model can be downloaded at https://huggingface.co/barneyhill/OligoAI. An interactive web interface for ASO design is available at https://sitlabs.org/OligoAI.

CONSORTIUM

UK Platform for Nucleic Acid Therapies (UPNAT): Alice Davidson (University College London), Nick Lench (MRC Harwell), Francesco Muntoni (University College London), Carlo Rinaldi (University of Oxford), Catherine Ryan (University College London), Mina Ryten (UK Dementia Research Institute, Cambridge University), Ella Francesca Whittle (University College London), Haiyan Zhou (University College London).

ACKNOWLEDGMENTS

This work was supported by a Wellcome Trust scholarship (218486/Z/19/Z) to B.H.; M.J., R.R.N., and P.L.O. were supported by an MRC program grant (MC_PC_20061). N.W. was supported by a Wellcome Career Development award (305292/Z/23/Z) and a Lister Institute research prize. A.C.H. was supported by the MRC grant TransNAT (MR/X008029/1). This work was also supported by the MRC Centre of Research Excellence in Therapeutic Genomics (MR/Z504725/1) to N.W., S.J.S., and C.R., the Health Data Research UK QQ2 Molecules to Health Records Driver Program to S.J.S., and the UK Platform for Nucleic Acid Therapies (UPNAT) (MR/Y008405/1) to C.R. C.R. was also supported by a Senior MRC fellowship (MR/Y008405/1).

AUTHOR CONTRIBUTIONS

Conceptualization, B.H., N.W., S.J.S., A.C.H., and C.R.; methodology, B.H., M.R.J., R.R.N., N.W., S.J.S., P.L.O., A.C.H., and C.R.; funding acquisition, B.H. and C.R.; project administration, N.W., S.J.S., P.L.O., A.C.H., and C.R.; supervision, N.W., S.J.S., P.L.O., A.C.H., and C.R.; writing – original draft, B.H.; writing – review and editing: N.W., M.J.A.W., S.J.S., P.L.O., A.C.H., and C.R.

DECLARATION OF INTERESTS

The authors declare no competing interests.

SUPPLEMENTAL INFORMATION

Supplemental information can be found online at https://doi.org/10.1016/j.omtn.2026.103004.

REFERENCES

1. Crooke, S.T., Baker, B.F., Crooke, R.M., and Liang, X.-H. (2021). Antisense technology: an overview and prospectus. Nat. Rev. Drug Discov. *20*, 427–453. https://doi.org/10.1038/s41573-021-00162-z.
2. Liang, X.-H., Sun, H., Nichols, J.G., and Crooke, S.T. (2017). RNase H1-Dependent Antisense Oligonucleotides Are Robustly Active in Directing RNA Cleavage in Both the Cytoplasm and the Nucleus. Mol. Ther. *25*, 2075–2092. https://doi.org/10.1016/j.ymthe.2017.06.002.
3. Crooke, S.T. (2017). Molecular Mechanisms of Antisense Oligonucleotides. Nucleic Acid Ther. *27*, 70–77. https://doi.org/10.1089/nat.2016.0656.
4. Egli, M., and Manoharan, M. (2023). Chemistry, structure and function of approved oligonucleotide therapeutics. Nucleic Acids Res. *51*, 2529–2573. https://doi.org/10.1093/nar/gkad067.
5. Khvorova, A., and Watts, J.K. (2017). The chemical evolution of oligonucleotide therapies of clinical utility. Nat. Biotechnol. *35*, 238–248. https://doi.org/10.1038/nbt.3765.
6. Zamecnik, P.C., and Stephenson, M.L. (1978). Inhibition of Rous sarcoma virus replication and cell transformation by a specific oligodeoxynucleotide. Proc. Natl. Acad. Sci. USA *75*, 280–284. https://doi.org/10.1073/pnas.75.1.280.
7. Paik, J., and Duggan, S. (2019). Volanesorsen: First Global Approval. Drugs *79*, 1349–1354. https://doi.org/10.1007/s40265-019-01168-z.
8. Keam, S.J. (2018). Inotersen: First Global Approval. Drugs *78*, 1371–1376. https://doi.org/10.1007/s40265-018-0968-5.
9. Crooke, S.T., Baker, B.F., Witztum, J.L., Kwoh, T.J., Pham, N.C., Salgado, N., McEvoy, B.W., Cheng, W., Hughes, S.G., Bhanot, S., and Geary, R.S. (2017). The Effects of 2’-O-Methoxyethyl Containing Antisense Oligonucleotides on Platelets in Human Clinical Trials. Nucleic Acid Ther. *27*, 121–129. https://doi.org/10.1089/nat.2016.0650.
10. Baker, B.F., Xia, S., Partridge, W., Engelhardt, J.A., Tsimikas, S., Crooke, S.T., Bhanot, S., and Geary, R.S. (2024). Safety and Tolerability of GalNAc3-Conjugated Antisense Drugs Compared to the Same-Sequence 2’-O-Methoxyethyl-Modified Antisense Drugs: Results from an Integrated Assessment of Phase 1 Clinical Trial Data. Nucleic Acid Ther. *34*, 18–25. https://doi.org/10.1089/nat.2023.0026.
11. Kim, J., Hu, C., Moufawad El Achkar, C., Black, L.E., Douville, J., Larson, A., Pendergast, M.K., Goldkind, S.F., Lee, E.A., Kuniholm, A., et al. (2019). Patient-Customized Oligonucleotide Therapy for a Rare Genetic Disease. N. Engl. J. Med. Overseas. Ed. *381*, 1644–1652. https://doi.org/10.1056/NEJMoa1813279.
12. Hwang, G., Kwon, M., Seo, D., Kim, D.H., Lee, D., Lee, K., Kim, E., Kang, M., and Ryu, J.-H. (2024). ASOptimizer: Optimizing antisense oligonucleotides through

deep learning for IDO1 gene regulation. Mol. Ther. Nucleic Acids *35*, 102186. https://doi.org/10.1016/j.omtn.2024.102186.

13. Skoufos, G., Kakoulidis, P., Tastsoglou, S., Zacharopoulou, E., Kotsira, V., Miliotis, M., Mavromati, G., Grigoriadis, D., Zioga, M., Velli, A., et al. (2024). TarBase-v9.0 extends experimentally supported miRNA–gene interactions to cell-types and virally encoded miRNAs. Nucleic Acids Res. *52*, D304–D310. https://doi.org/10.1093/nar/gkad1071.

14. Gresova, K., Racek, T., Martinek, V., Cechak, D., Svobodova, R., and Alexiou, P. (2023). RBP-Tar – a searchable database for experimental RBP binding sites. F1000Res. *12*, 755. https://doi.org/10.12688/f1000research.131014.3.

15. Lorenz, R., Bernhart, S.H., Höner zu Siederdissen, C., Tafer, H., Flamm, C., Stadler, P.F., and Hofacker, I.L. (2011). ViennaRNA Package 2.0. Algorithms Mol. Biol. *6*, 26. https://doi.org/10.1186/1748-7188-6-26.

16. Mayya, V.K., and Duchaine, T.F. (2019). Ciphers and Executioners: How 3′-Untranslated Regions Determine the Fate of Messenger RNAs. Front. Genet. *10*, 6. https://doi.org/10.3389/fgene.2019.00006.

17. Matveeva, O.V., Tsodikov, A.D., Giddings, M., Freier, S.M., Wyatt, J.R., Spiridonov, A.N., Shabalina, S.A., Gesteland, R.F., and Atkins, J.F. (2000). Identification of sequence motifs in oligonucleotides whose presence is correlated with antisense activity. Nucleic Acids Res. *28*, 2862–2865. https://doi.org/10.1093/nar/28.15.2862.

18. Penić, R.J., Vlašić, T., Huber, R.G., Wan, Y., and Šikić, M. (2025). RiNALMo: General-Purpose RNA Language Models Can Generalize Well on Structure Prediction Tasks. Nat. Commun. *16*, 5671. https://doi.org/10.1038/s41467-025-60872-5.

19. Lu, Z.J., and Mathews, D.H. (2008). Efficient siRNA selection using hybridization thermodynamics. Nucleic Acids Res. *36*, 640–647. https://doi.org/10.1093/nar/gkm920.

20. Barcia, G., Fleming, M.R., Deligniere, A., Gazula, V.-R., Brown, M.R., Langouet, M., Chen, H., Kronengold, J., Abhyankar, A., Cilio, R., et al. (2012). De novo gain-of-function KCNT1 channel mutations cause malignant migrating partial seizures of infancy. Nat. Genet. *44*, 1255–1259. https://doi.org/10.1038/ng.2441.

21. Heron, S.E., Smith, K.R., Bahlo, M., Nobili, L., Kahana, E., Licchetta, L., Oliver, K.L., Mazarib, A., Afawi, Z., Korczyn, A., et al. (2012). Missense mutations in the sodium-gated potassium channel gene KCNT1 cause severe autosomal dominant nocturnal frontal lobe epilepsy. Nat. Genet. *44*, 1188–1190. https://doi.org/10.1038/ng.2440.

22. Long, D., Lee, R., Williams, P., Chan, C.Y., Ambros, V., and Ding, Y. (2007). Potent effect of target structure on microRNA function. Nat. Struct. Mol. Biol. *14*, 287–294. https://doi.org/10.1038/nsmb1226.

23. Ho, S.P., Bao, Y., Lesher, T., Malhotra, R., Ma, L.Y., Fluharty, S.J., and Sakai, R.R. (1998). Mapping of RNA accessible sites for antisense experiments with oligonucleotide libraries. Nat. Biotechnol. *16*, 59–63. https://doi.org/10.1038/nbt0198-59.

24. Shao, Y., Wu, Y., Chan, C.Y., McDonough, K., and Ding, Y. (2006). Rational design and rapid screening of antisense oligonucleotides for prokaryotic gene modulation. Nucleic Acids Res. *34*, 5660–5669. https://doi.org/10.1093/nar/gkl715.

25. Means, J.C., Martinez-Bengochea, A.L., Louiselle, D.A., Nemechek, J.M., Perry, J.M., Farrow, E.G., Pastinen, T., and Younger, S.T. (2025). Rapid and scalable personalized ASO screening in patient-derived organoids. Nature *638*, 237–243. https://doi.org/10.1038/s41586-024-08462-1.

26. Singh, A., Fry, A., Perelman, A., Tart, A., Ganesh, A., El-Kishky, A., McLaughlin, A., Low, A., Ostrow, A.J., Ananthram, A., et al. (2025). OpenAI GPT-5 System Card. Preprint at arXiv. https://doi.org/10.48550/arXiv.2601.03267.

27. Dyer, S.C., Austine-Orimoloye, O., Azov, A.G., Barba, M., Barnes, I., Barrera-Enriquez, V.P., Becker, A., Bennett, R., Beracochea, M., Berry, A., et al. (2025). Ensembl 2025. Nucleic Acids Res. *53*, D948–D957. https://doi.org/10.1093/nar/gkae1071.

28. Virtanen, P., Gommers, R., Oliphant, T.E., Haberland, M., Reddy, T., Cournapeau, D., Burovski, E., Peterson, P., Weckesser, W., Bright, J., et al. (2020). SciPy 1.0: fundamental algorithms for scientific computing in Python. Nat. Methods *17*, 261–272. https://doi.org/10.1038/s41592-019-0686-2.

29. Zuker, M. (2003). Mfold web server for nucleic acid folding and hybridization prediction. Nucleic Acids Res. *31*, 3406–3415. https://doi.org/10.1093/nar/gkg595.

30. Lange, S.J., Maticzka, D., Möhl, M., Gagnon, J.N., Brown, C.M., and Backofen, R. (2012). Global or local? Predicting secondary structure and accessibility in mRNAs. Nucleic Acids Res. *40*, 5215–5226. https://doi.org/10.1093/nar/gks181.

31. Srivastava, N., Hinton, G., Krizhevsky, A., Sutskever, I., and Salakhutdinov, R. (2014). Dropout: A Simple Way to Prevent Neural Networks from Overfitting. J. Mach. Learn. Res. *15*, 1929–1958.

32. Kingma, D.P., and Ba, J. (2017). Adam: A Method for Stochastic Optimization. Preprint at arXiv. https://doi.org/10.48550/arXiv.1412.6980.

33. John, B., Enright, A.J., Aravin, A., Tuschl, T., Sander, C., and Marks, D.S. (2004). Human MicroRNA Targets. PLoS Biol. *2*, e363. https://doi.org/10.1371/journal.pbio.0020363.

OMTN, Volume *37*

Supplemental information

Accurately modeling RNase H-mediated antisense oligonucleotide efficacy

Barney Hill, Maisie R. Jaques, Remya R. Nair, Nicola Whiffin, Matthew J.A. Wood, Stephan J. Sanders, Peter L. Oliver, Alyssa C. Hill, Carlo Rinaldi, and on behalf of the UPNAT Consortium

Supplemental Material

Supplemental Text

LLM Prompts for Data Extraction

To ensure the reproducibility of our data extraction pipeline, this section details the verbatim prompts used for table normalisation with gpt-5.

```
Prompt 1

## Task
Write a Python 3.11 script to convert OCR-extracted table XML data into a
structured CSV format.

## Output Format
Return a Script object with:
- `pyscript`: Complete Python conversion script as a string

## Function Requirements
- The Python script should contain a function `xml_to_csv(xml_str: str) ->
str`
- You do need to return if __name__ ... w/ example usage - just our
function.

## Technical Requirements
### Dependencies
- Uses only Python standard library + `re` module

### Column Name Rules
- Preserve meaning of original column headers in new csv. i.e "UTC
Untreated control group (%)" to "utc_untreated_control_group_pct" /
"Inhibition (%)" to "inhibition_pct"
- Make SQL-compatible: underscores for spaces, no dots, lowercase
- Hardcode column names (no need to dynamically generate)

### Data Handling
- Use "NA" for missing/empty cells
- Properly escape CSV values (quotes, commas, newlines)
- Use comma delimiter
- Sometimes   is used in the XML, this should be replaced with a
space in the CSV.

## Domain Context
- UTC = "Untreated Control" percentage - this is not inhibition
- Preserve scientific notation and decimal precision
- Don't interpret abbreviations unless obvious

## Quality control
- To ensure correct rows let's strip newspace and capitalise the sequence
column. It should have >=8 ATGC characters. If not skip the row.
```

```
## Preview of the input XML Structure (xml_str) - we will use the full
version as input for your script (do not return this in your output!):
Your goal is to produce a dataset of antisense-oligonucleotide sequences
and their inhibition percentages.
To do so you must stack the secondary_table with the primary_table using a
SQL command.

Required Columns in secondary_table:
1. ASO sequence (case insensitive)
2. One of:
   - inhibition/knockdown/reduction percentage
   -  UTC (Untreated Control) / RNA percentage

Transformation Rules:
- Numeric columns -> DOUBLE
- inhibition_percent =
    - Direct copy from inhibition/knockdown columns
    - 100 - UTC(%) for untreated control
- CONCAT two columns if ASO sequence is split (e.g.,sequence_part_one,
sequence_part_two)
- When using CAST be careful, some rows may not be castable to double,
hence use TRY_CAST.

Task:
1. Generate SQL:
   - Stack (INSERT INTO) secondary_table onto primary_table
   - Apply transformations as needed
   - **CRITICAL: Always use "secondary_table" as the table name in your
FROM clause**
   - **When referencing columns from secondary_table, use the exact column
names shown in the schema, including any special characters or numbers.**

Output Format:
- sql_command: string containing complete SQL command to stack the
secondary_table onto primary_table

Data:
primary_table:
Schema:
- aso_sequence_5_to_3 (VARCHAR): 5'-3' ASO nucleotide sequence
- inhibition_percent (DOUBLE): target inhibition percentage, range 0-100
```

Prompt 2

```
Your goal is to produce a dataset of antisense-oligonucleotide sequences
and their inhibition percentages.
To do so you must stack the secondary_table with the primary_table using a
SQL command.

Required Columns in secondary_table:
1. ASO sequence (case insensitive)
2. One of:
   - inhibition/knockdown/reduction percentage
   -  UTC (Untreated Control) / RNA percentage
```

```
Transformation Rules:
- Numeric columns -> DOUBLE
- inhibition_percent =
    - Direct copy from inhibition/knockdown columns
    - 100 - UTC(%) for untreated control
- CONCAT two columns if ASO sequence is split (e.g.,sequence_part_one,
sequence_part_two)
- When using CAST be careful, some rows may not be castable to double,
hence use TRY_CAST.

Task:
1. Generate SQL:
   - Stack (INSERT INTO) secondary_table onto primary_table
   - Apply transformations as needed
   - **CRITICAL: Always use "secondary_table" as the table name in your
FROM clause**
   - **When referencing columns from secondary_table, use the exact column
names shown in the schema, including any special characters or numbers.**

Output Format:
- sql_command: string containing complete SQL command to stack the
secondary_table onto primary_table

Data:
primary_table:
Schema:
- aso_sequence_5_to_3 (VARCHAR): 5'-3' ASO nucleotide sequence
- inhibition_percent (DOUBLE): target inhibition percentage, range 0-100
```

Supplemental Tables

Table S1. Description of the fields, data types, and definitions for each entry in the ASO Atlas dataset.

Field Name	Data Type	Description
aso_sequence_5_to_3	String	The nucleotide sequence of the ASO.
inhibition_percent	Float	The measured percentage of target RNA inhibition. Typically 0-100 although there are some values < 0 where upregulation was observed.
chemistry	Object	A object containing a list of all sugar and backbone modifications
custom_id	String	A string refering to the location of the referenced patent table file.
target_mrna	String	The name used to refer to the target mRNA.
target_gene	String	The HUGO gene name corresponding the the target_mrna
cell_line	String	The cell-line used for the screen.
dosage	Float	The dosage of the administered ASO in nM.
cells_per_well	Integer	The number of cells per well used in the screen.

Field Name	Data Type	Description
transfection_method	String	The method of introducing the ASO into the cells.

Supplemental Figures

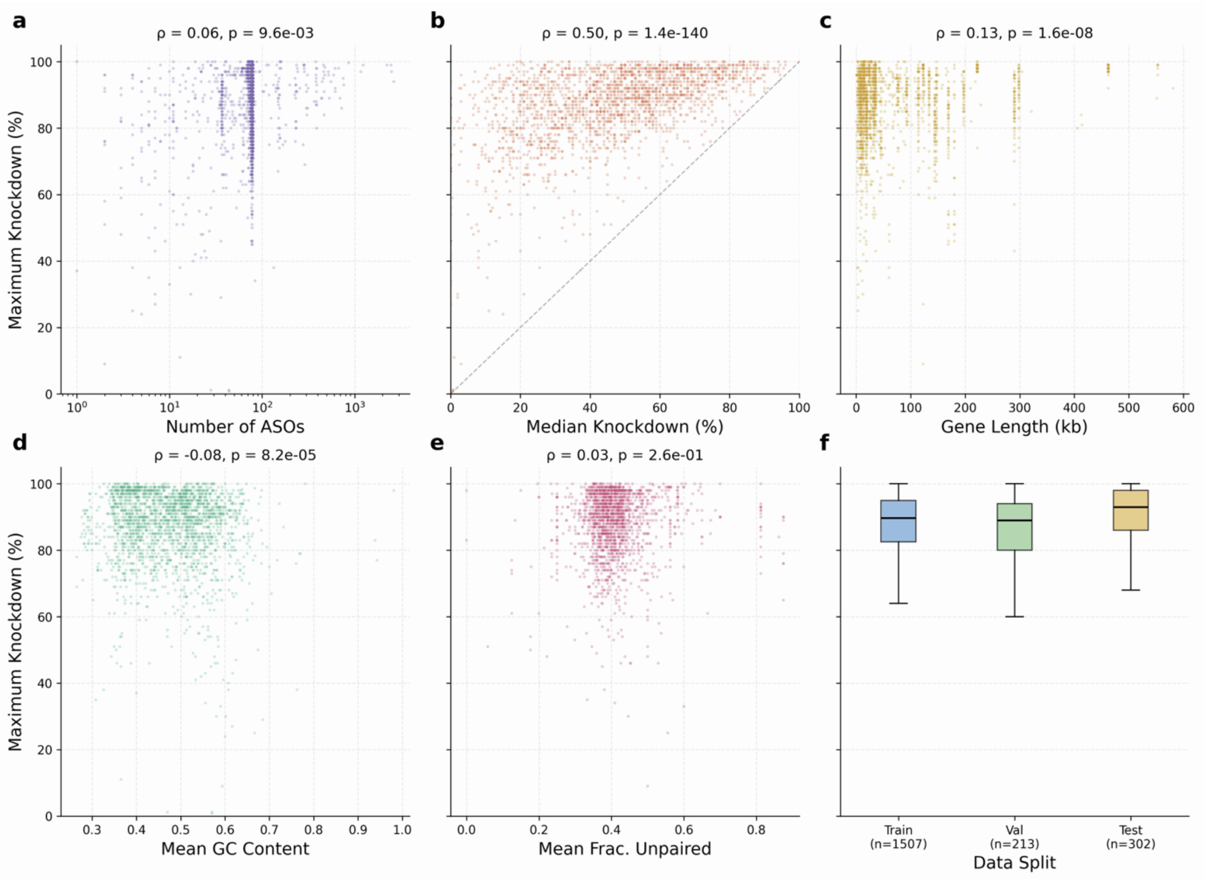

Figure S1. Influence of screen size, target properties, and data partitioning on maximum knockdown per screen. (a) Maximum knockdown as a function of the number of ASOs per screen (Spearman $\rho = 0.06, p = 9.6 \times 10^{-3}$). (b) Maximum versus median knockdown per screen ($\rho = 0.50, p = 1.4 \times 10^{-140}$). (c) Maximum knockdown versus gene length (genomic span; $\rho = 0.13, p = 1.6 \times 10^{-8}$). (d) Maximum knockdown versus mean ASO GC content ($\rho = -0.08, p = 8.2 \times 10^{-5}$). (e) Maximum knockdown versus mean fraction of unpaired nucleotides at the target site ($\rho = 0.03, p = 0.26$). (f) Distribution of maximum knockdown across training, validation, and test splits. Each point in (a–e) represents one screen. All correlations are Spearman rank coefficients.

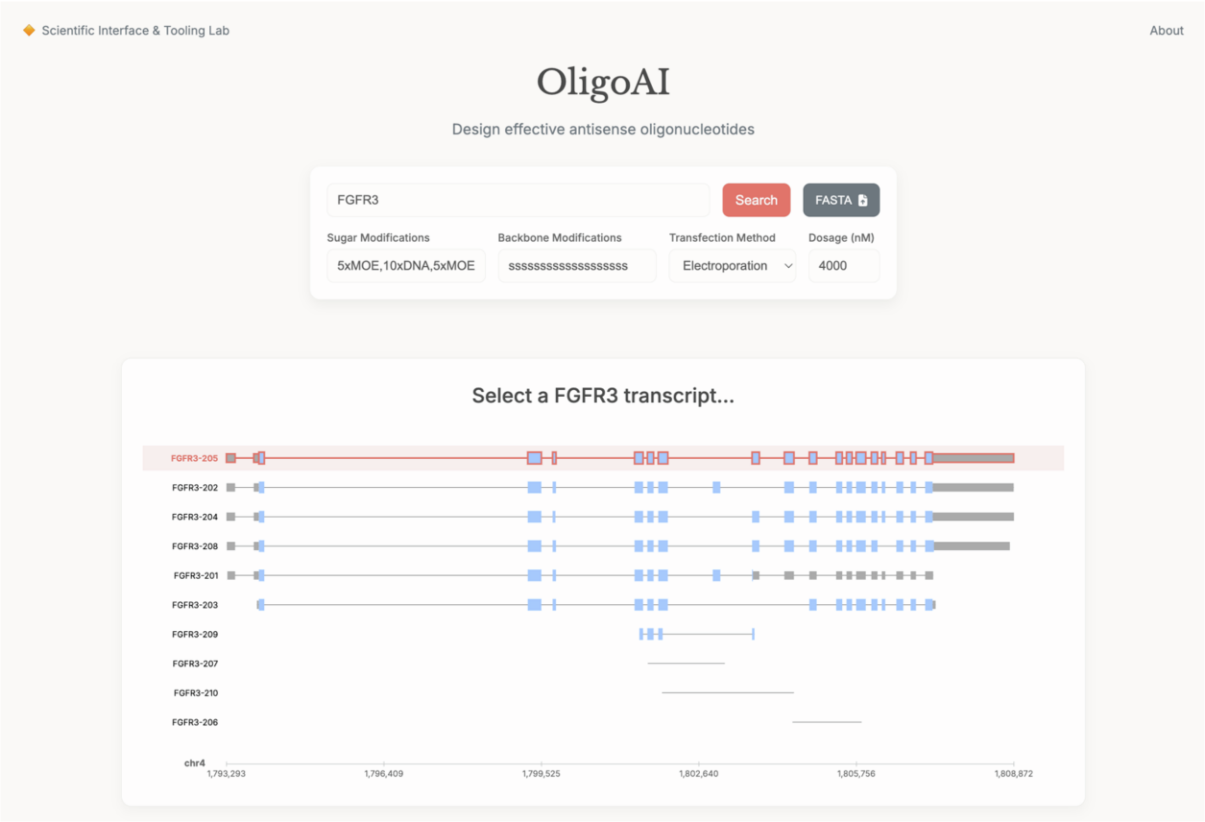

Figure S2. OligoAI online portal. Users can either supply a target RNA via FASTA file upload or select an Ensembl human transcript. When a target is specified, the OligoAI model processes the target RNA using serverless GPU inference. These results are returned to the user on the same page or via an email reminder and are available to download.

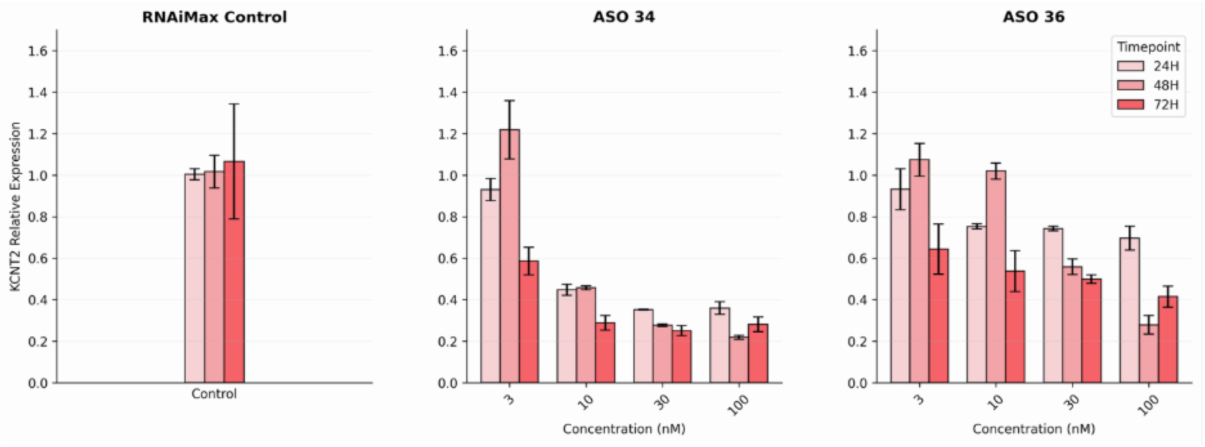

Figure S3. Dose- and time-dependent knockdown of *KCNT2* expression by selected ASOs in HeLa cells. Individual panels show the relative activity for three ASOs. Cells were treated at concentrations of 3, 10, 30 and 100 nM for 24, 48 or 72 hours. *KCNT2* mRNA levels were measured by quantitative RT-PCR and normalised to *HPRT*. Data represent mean $\pm$ SEM of three biological replicates per condition. Based on these results, 30 nM treatment for 48 hours was selected as the optimal condition. Data are shown relative to transfection reagent alone (RNAiMAX).

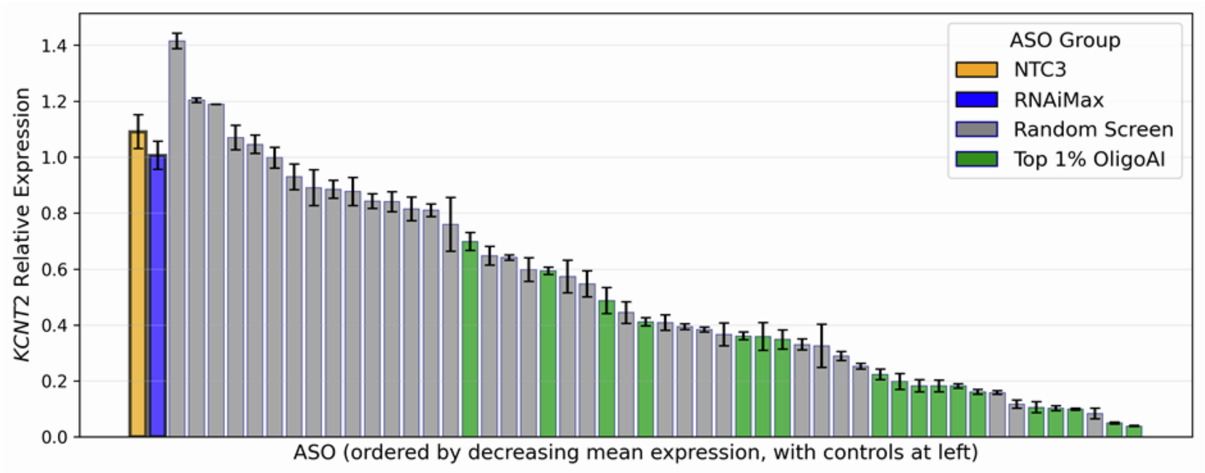

Figure S4. Bar plot showing relative *KCNT2* expression levels for all 50 screened ASOs ordered by decreasing mean expression (most effective knockdown on the right). HeLa cells were transfected with individual ASOs at 30 nM for 48 hours. Relative gene expression was determined by qRT-PCR with *HPRT* as the reference gene. Error bars represent SEM from three biological replicates. Bars are colored by ASO selection group: randomly selected ASOs (grey bars, standard screen, $N = 32$) and ASOs from the top 1% of OligoAI scores (green bars, predicted active, $N = 18$). The distribution demonstrates that OligoAI predictions correlate with experimental efficacy, with most top 1% ASOs showing greater knockdown activity (lower relative expression) compared to bottom 1% ASOs. Data are shown relative to transfection reagent alone (RNAiMAX) and a non-targeting control (NTC) ASO of matching chemical composition.

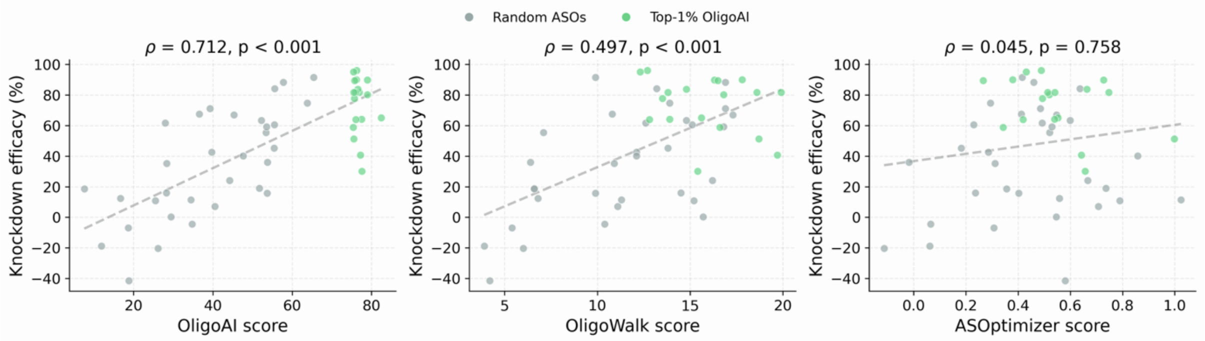

Figure S5. Correlation between predicted and measured *KCNT2* knockdown across methods. Scatter plots showing predicted score versus measured knockdown efficacy for all 50 experimentally tested *KCNT2* ASOs (32 randomly selected, 18 top-1% OligoAI-ranked), scored by OligoAI, OligoWalk, and ASOptimizer. Spearman rank correlation and associated *P*-values are shown above each panel. Trend lines are fitted by ordinary least squares.

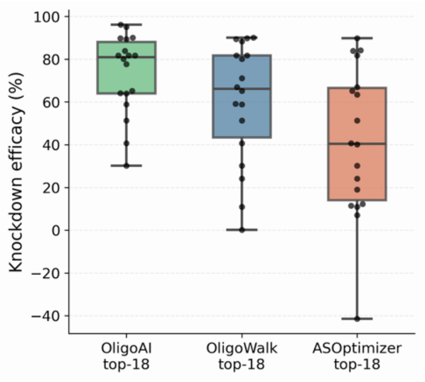

Figure S6. Comparison of top-18 ASO selections by each prediction method. Box-and-swarm plots showing the measured knockdown efficacy of the 18 ASOs each method would have prioritised from the 50 experimentally tested *KCNT2* ASOs. OligoAI's top-18 selections achieved the highest median knockdown (81%), compared with OligoWalk (66%) and ASOptimizer (40%).

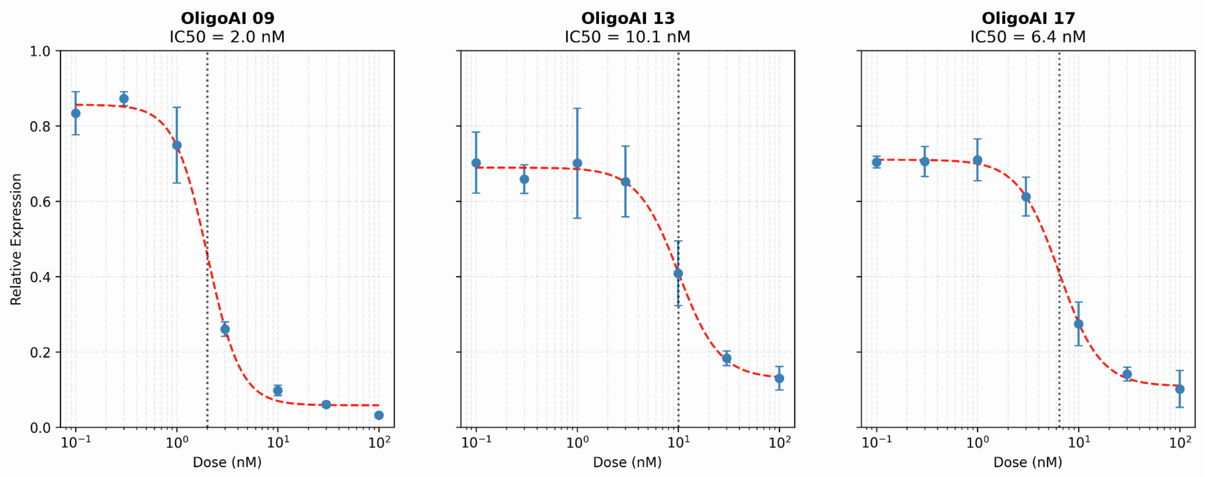

Figure S7. Individual dose-response curves showing relative *KCNT2* expression levels following ASO treatment in HeLa cells. Cells were transfected with ASOs at concentrations ranging from 0.1 nM to 1 μM and incubated for 48 hours. Each data point represents the mean $\pm$ SEM of three biological replicates. Relative gene expression was determined by qRT-PCR with *HPRT* as the reference gene. Red dashed lines show 4-parameter logistic curve fits (Hill equation). Black dotted vertical lines indicate calculated IC50 values. IC50 values demonstrate knockdown activity in the low nanomolar range.
